# Supplementary figures and images for: PM2.5 from automobile exhaust induces apoptosis in male rat germ cells via the ROS-UPR mt signaling pathway
Source: PLoS One. 2025 Apr 2;20(4):e0313803. doi: 10.1371/journal.pone.0313803 (PMC11964221; doi:10.1371/journal.pone.0313803)

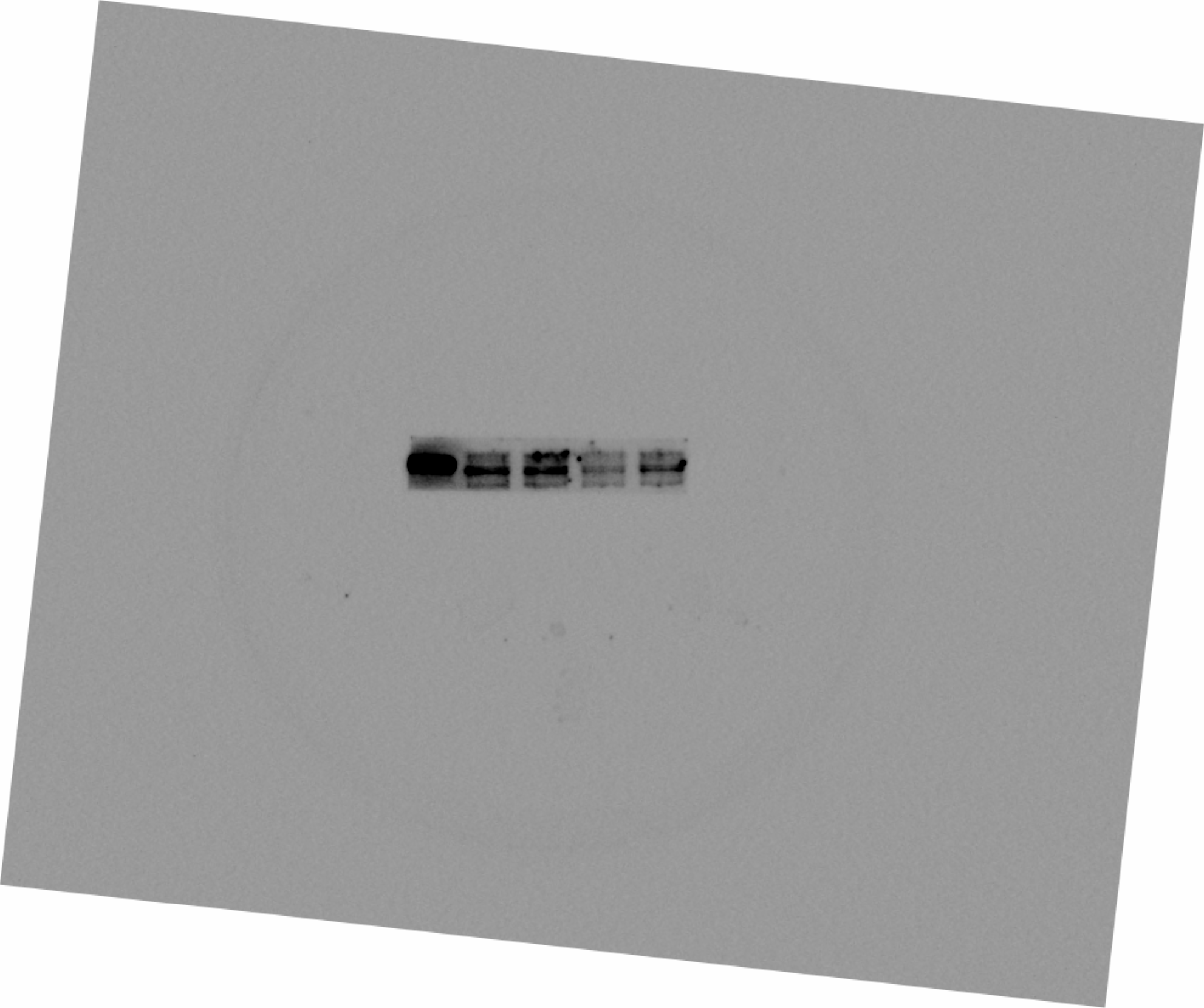

Supplement: S2 File — (ZIP) [file pone.0313803.s002.zip › Uncropped western blots/WB-ATF5/SYZX 2024-04-17 10h25m22s.tif]

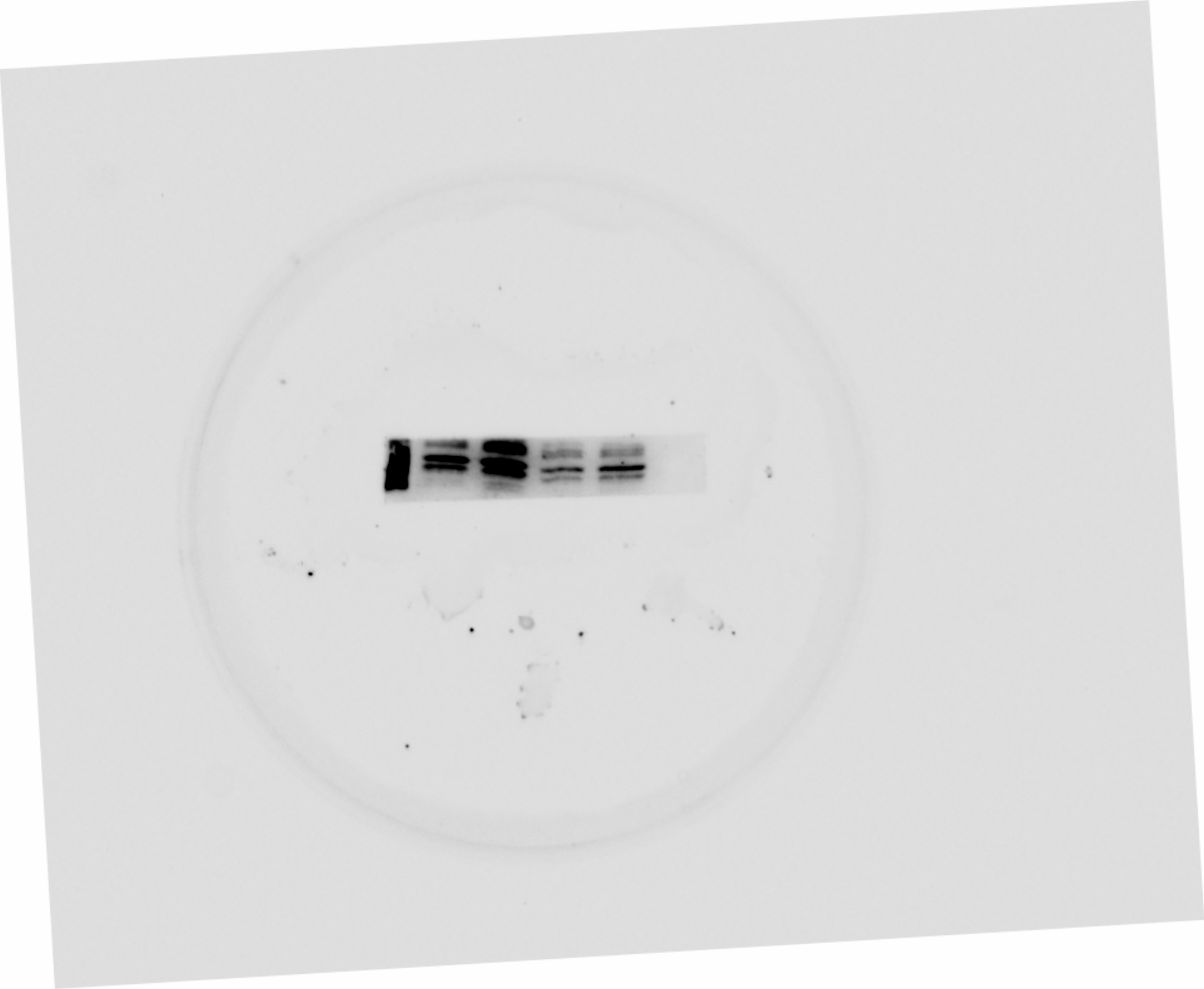

Supplement: S2 File — (ZIP) [file pone.0313803.s002.zip › Uncropped western blots/WB-ATF5/SYZX 2024-04-17 10h36m40s.tif]

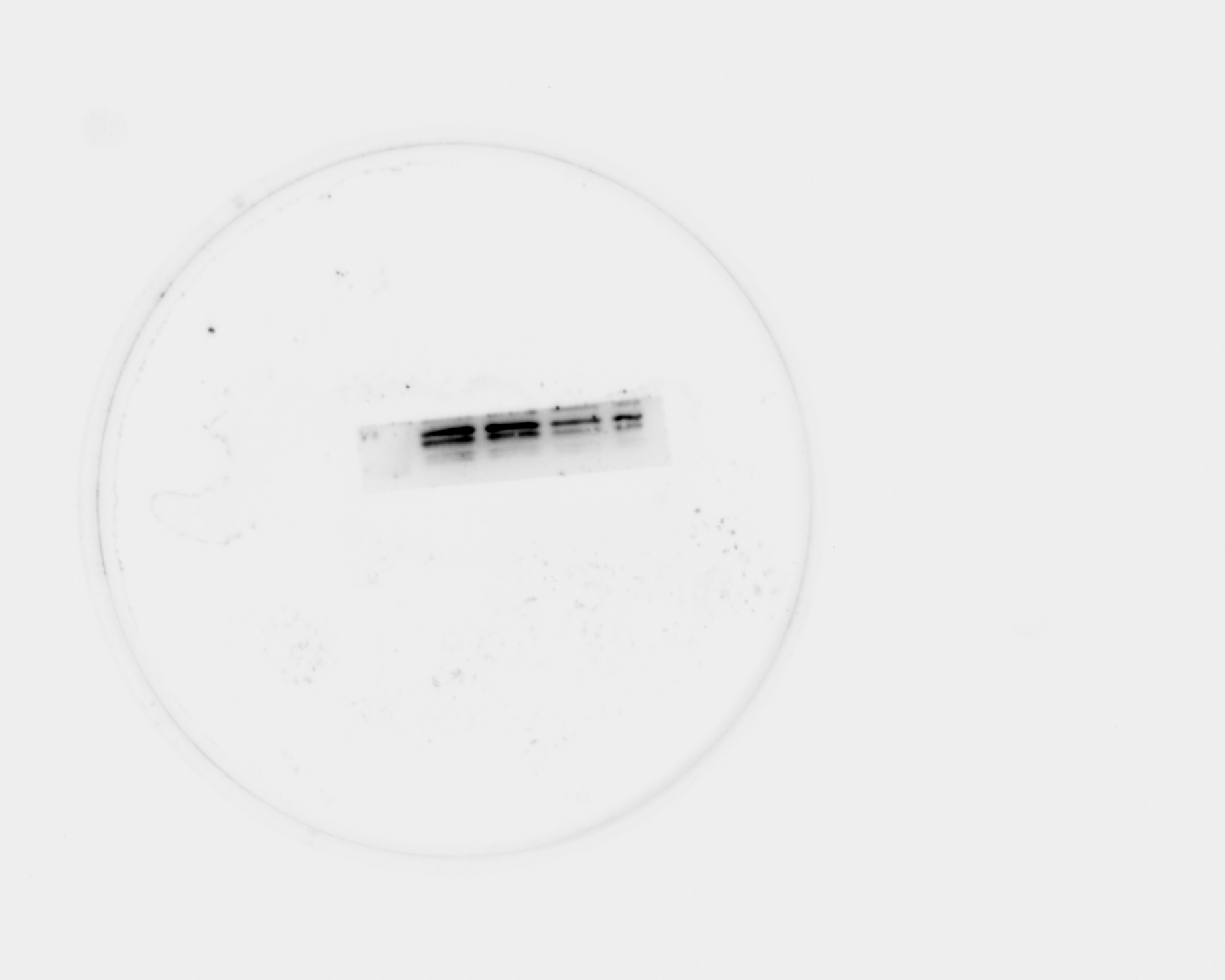

Supplement: S2 File — (ZIP) [file pone.0313803.s002.zip › Uncropped western blots/WB-ATF5/SYZX 2024-04-25 10h20m22s.tif]

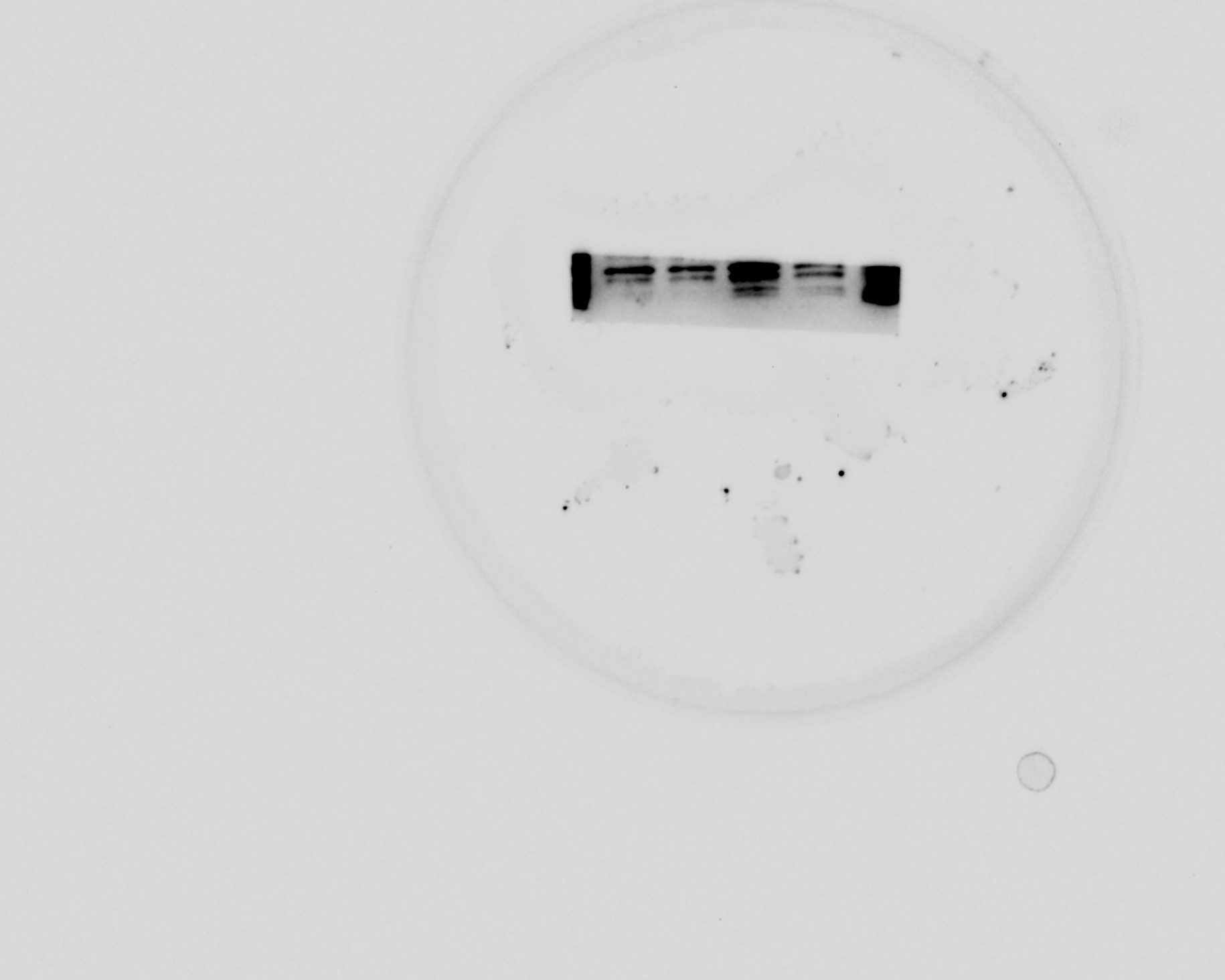

Supplement: S2 File — (ZIP) [file pone.0313803.s002.zip › Uncropped western blots/WB-Bax/SYZX 2024-04-17 10h34m37s.tif]

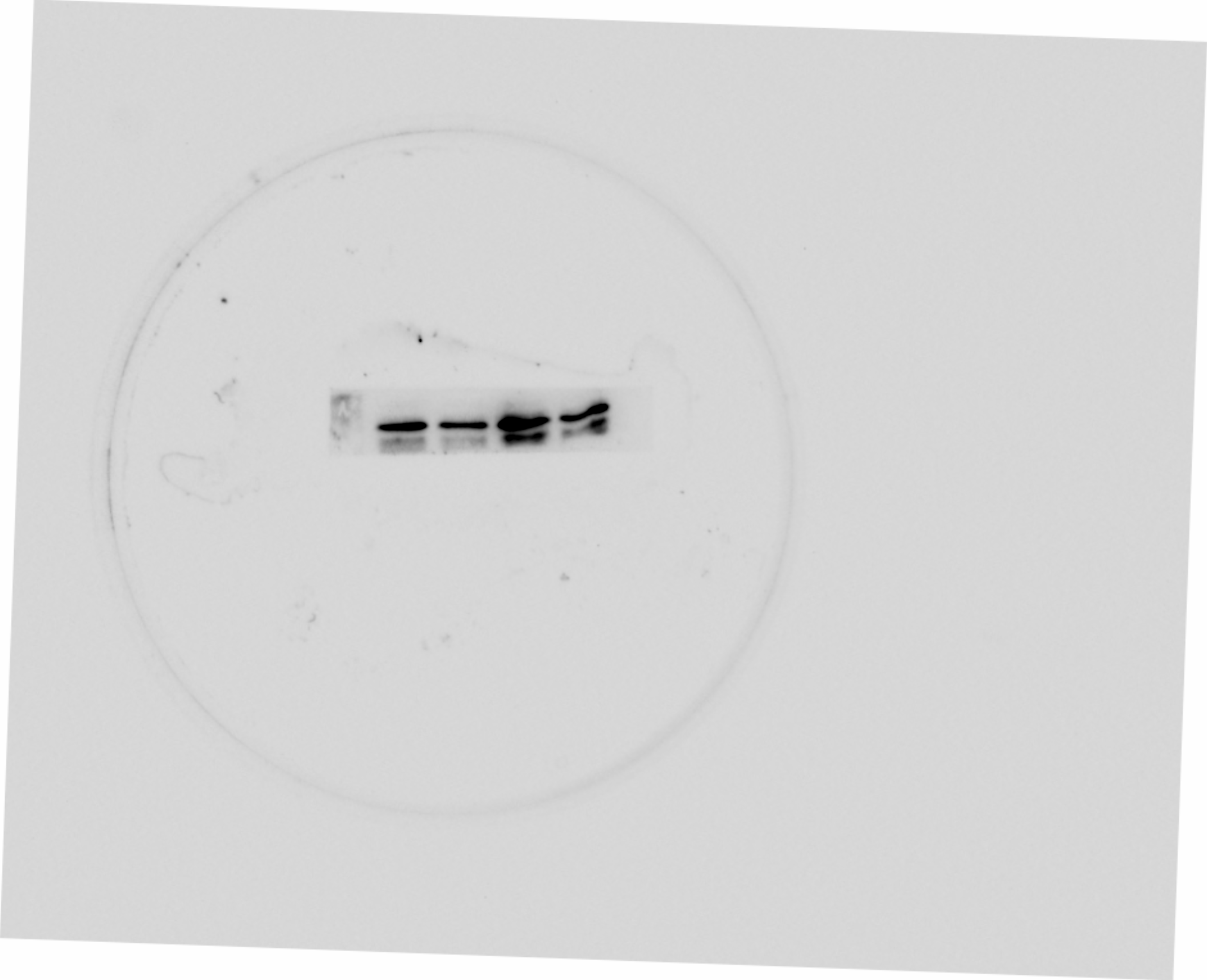

Supplement: S2 File — (ZIP) [file pone.0313803.s002.zip › Uncropped western blots/WB-Bax/SYZX 2024-04-18 12h5m10s.tif]

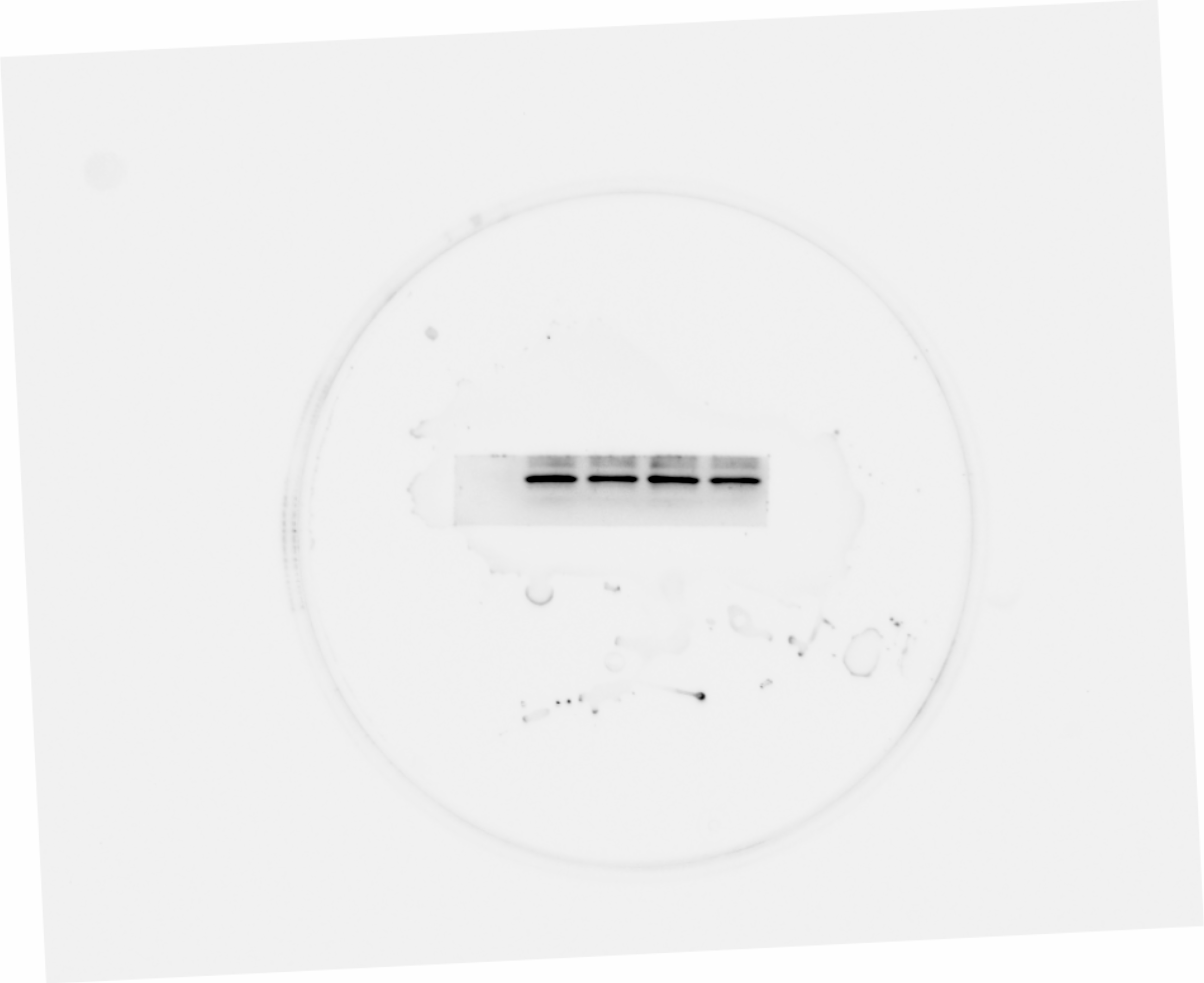

Supplement: S2 File — (ZIP) [file pone.0313803.s002.zip › Uncropped western blots/WB-Bax/SYZX 2024-04-25 12h28m22s.tif]

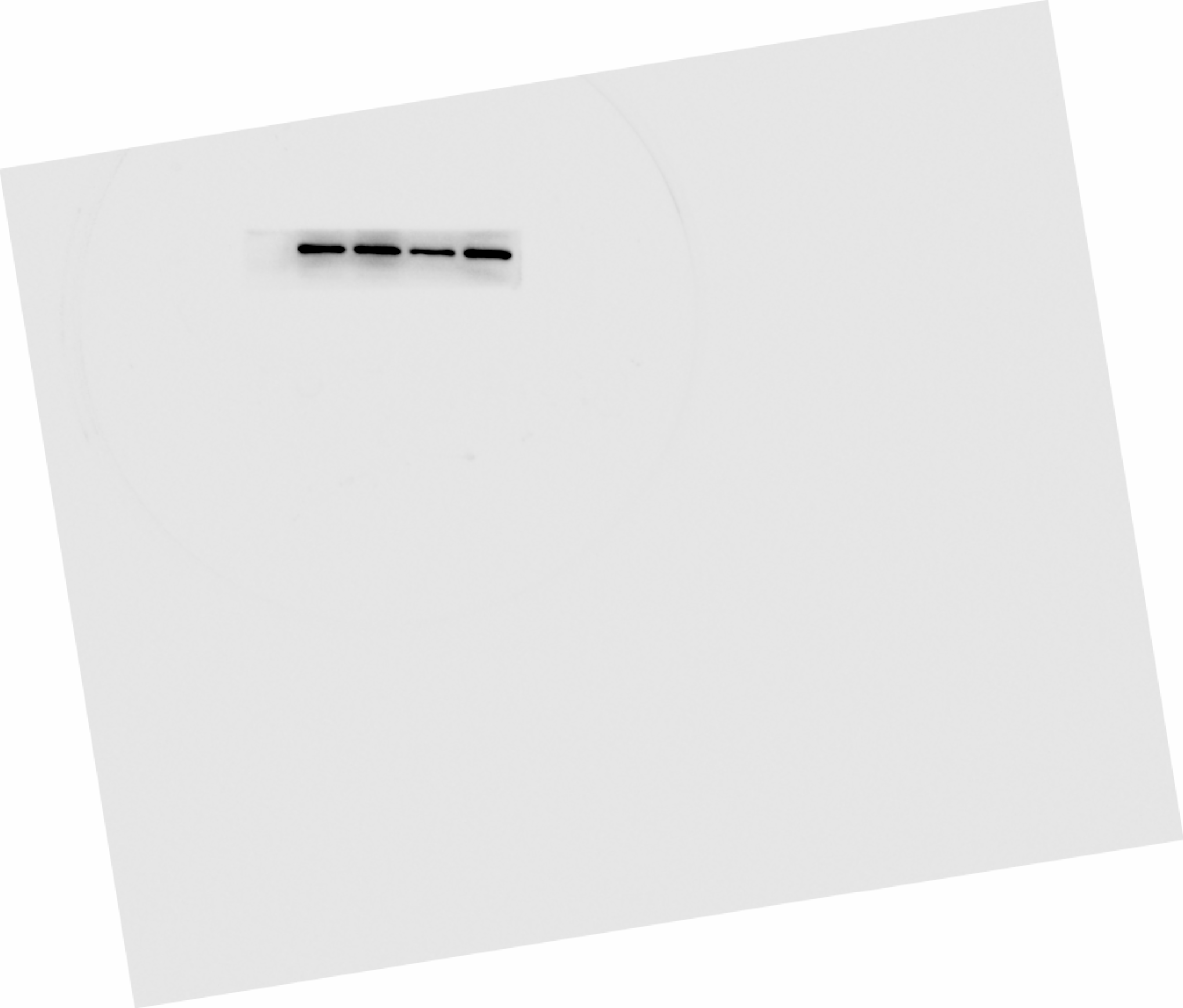

Supplement: S2 File — (ZIP) [file pone.0313803.s002.zip › Uncropped western blots/WB-Bcl2/SYZX 2024-04-18 11h56m18s.tif]

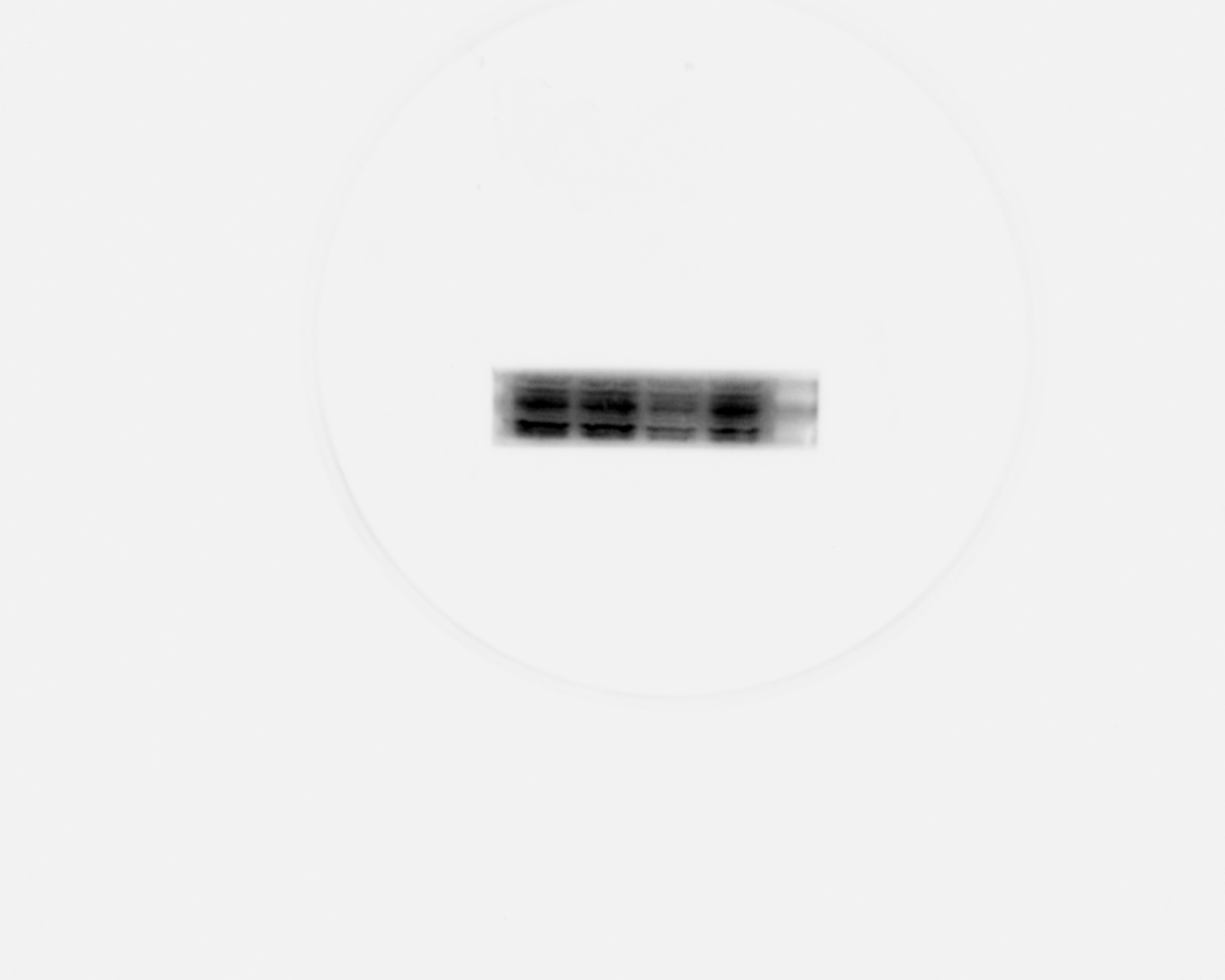

Supplement: S2 File — (ZIP) [file pone.0313803.s002.zip › Uncropped western blots/WB-Bcl2/SYZX 2024-04-25 12h12m48s.jpg]

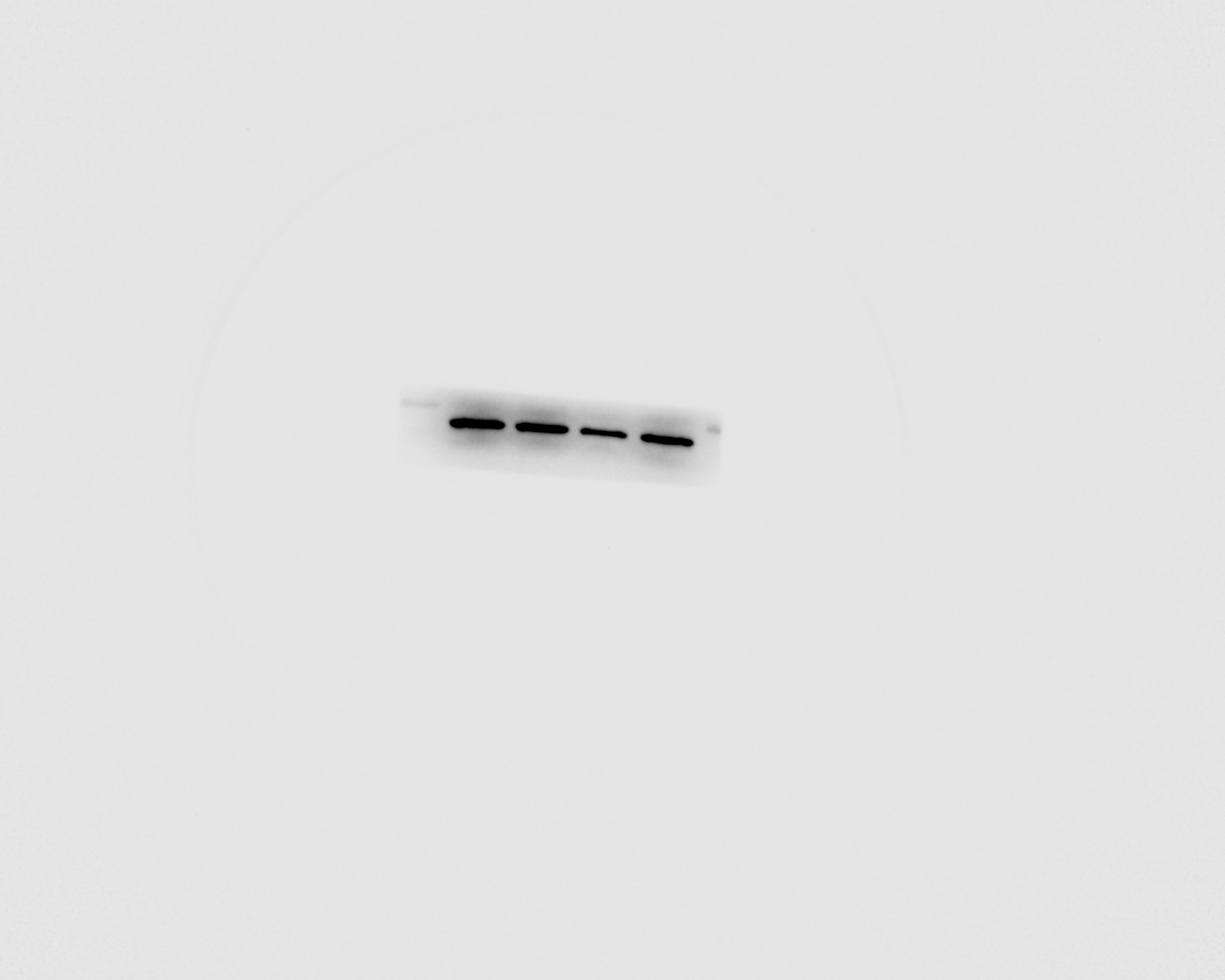

Supplement: S2 File — (ZIP) [file pone.0313803.s002.zip › Uncropped western blots/WB-Bcl2/SYZX 2024-04-25 12h28m22s.jpg]

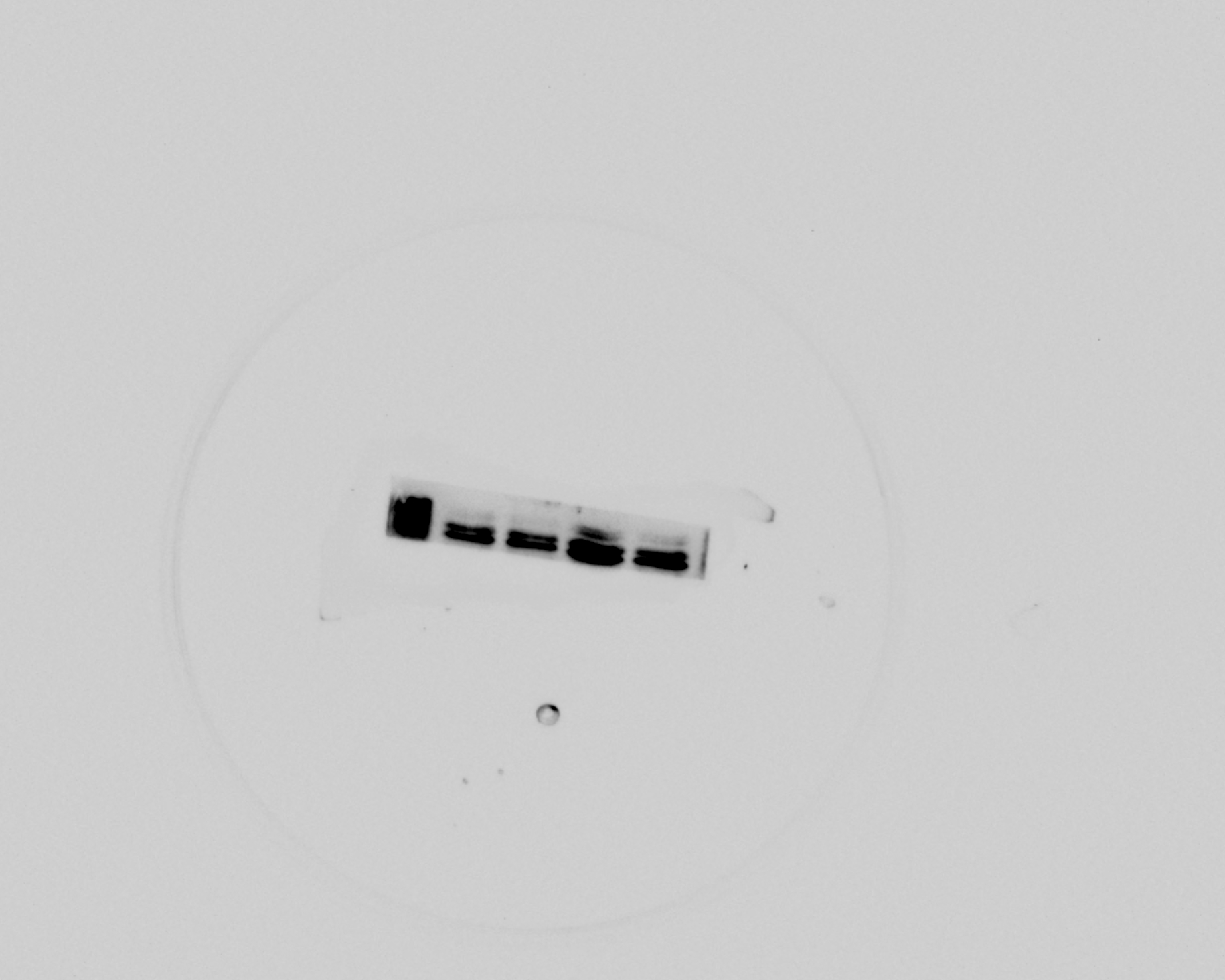

Supplement: S2 File — (ZIP) [file pone.0313803.s002.zip › Uncropped western blots/WB-MDA/SYZX 2024-04-17 10h41m43s.jpg]

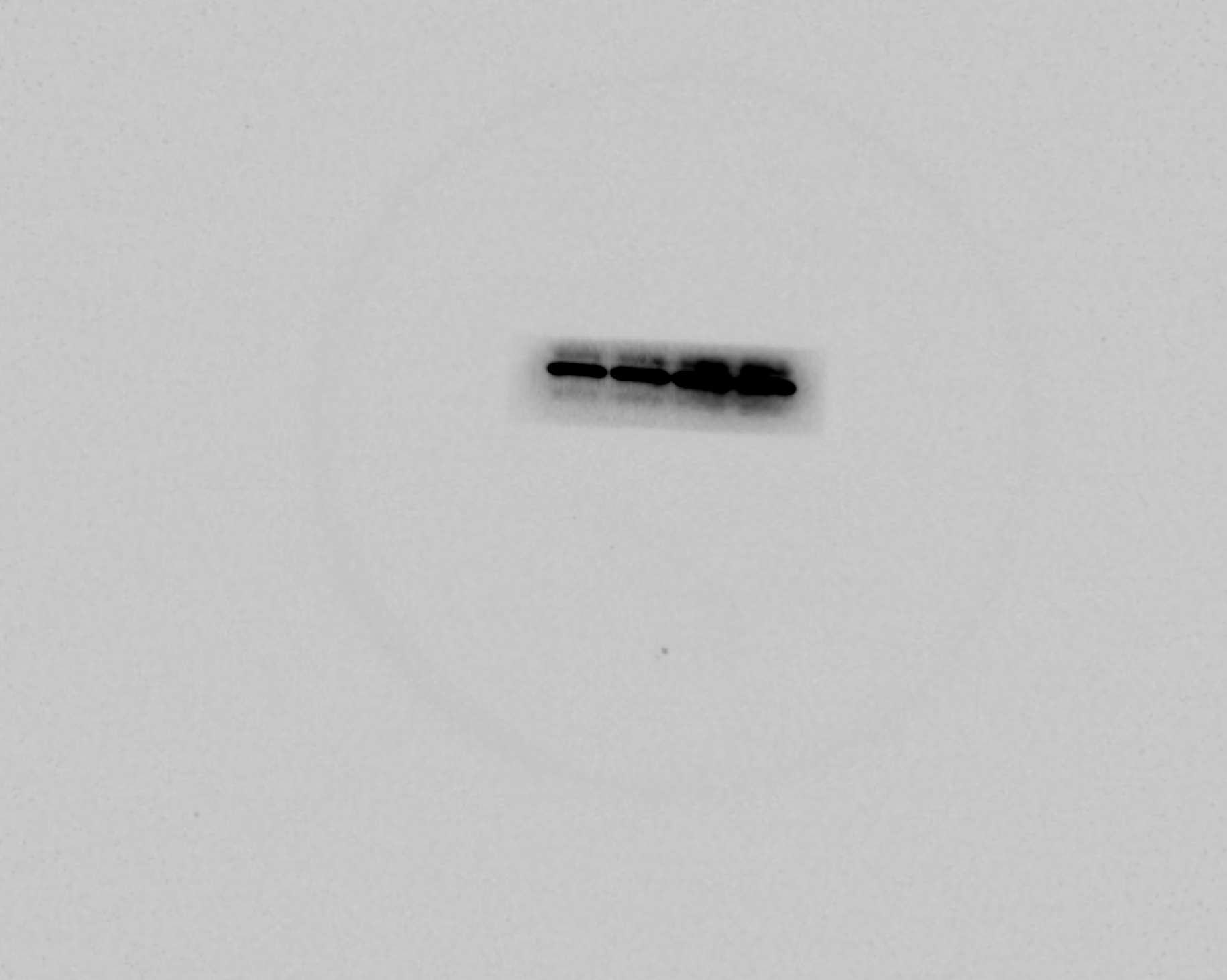

Supplement: S2 File — (ZIP) [file pone.0313803.s002.zip › Uncropped western blots/WB-MDA/SYZX 2024-04-18 11h56m59s.jpg]

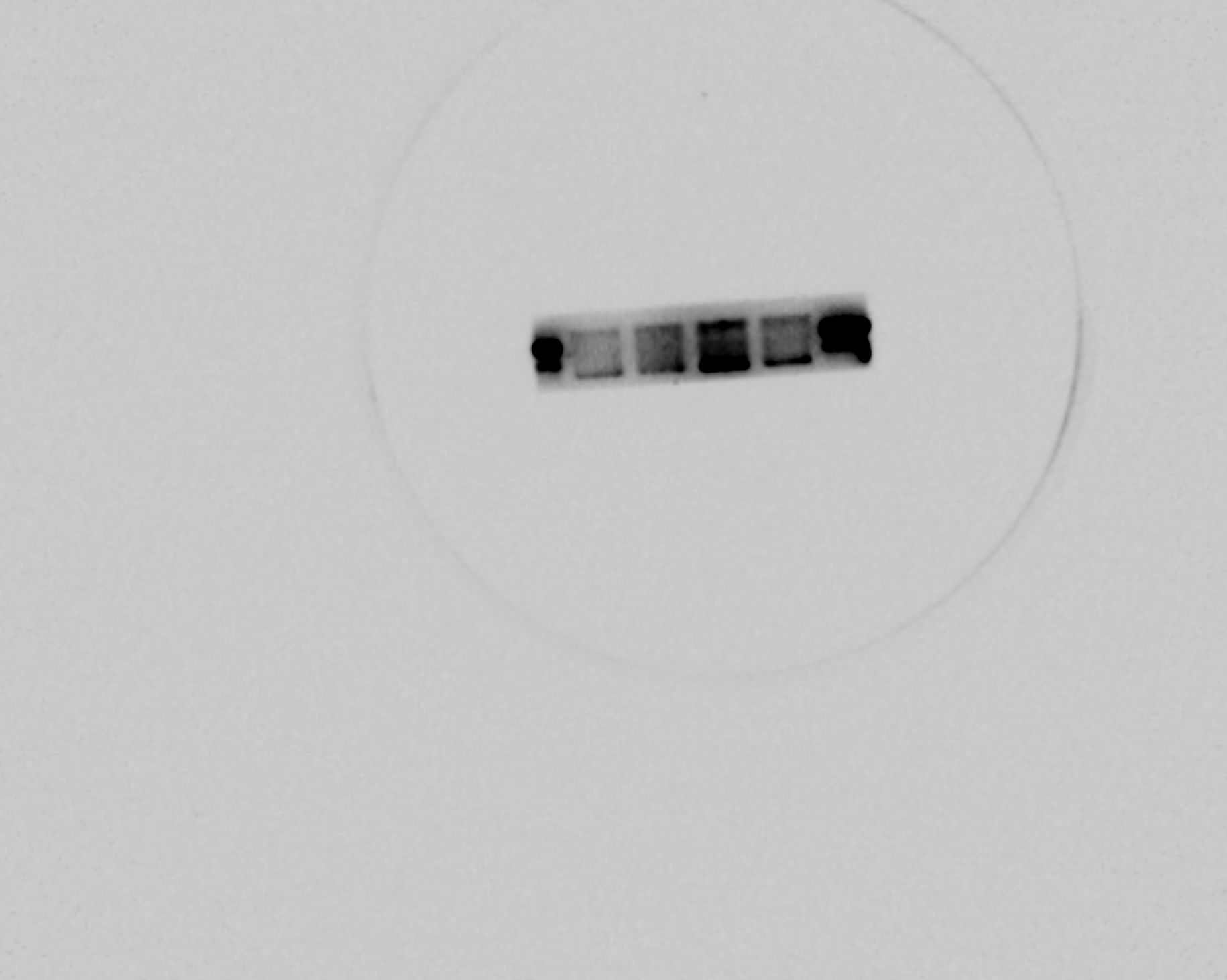

Supplement: S2 File — (ZIP) [file pone.0313803.s002.zip › Uncropped western blots/WB-MDA/SYZX 2024-04-18 12h03m56s.jpg]

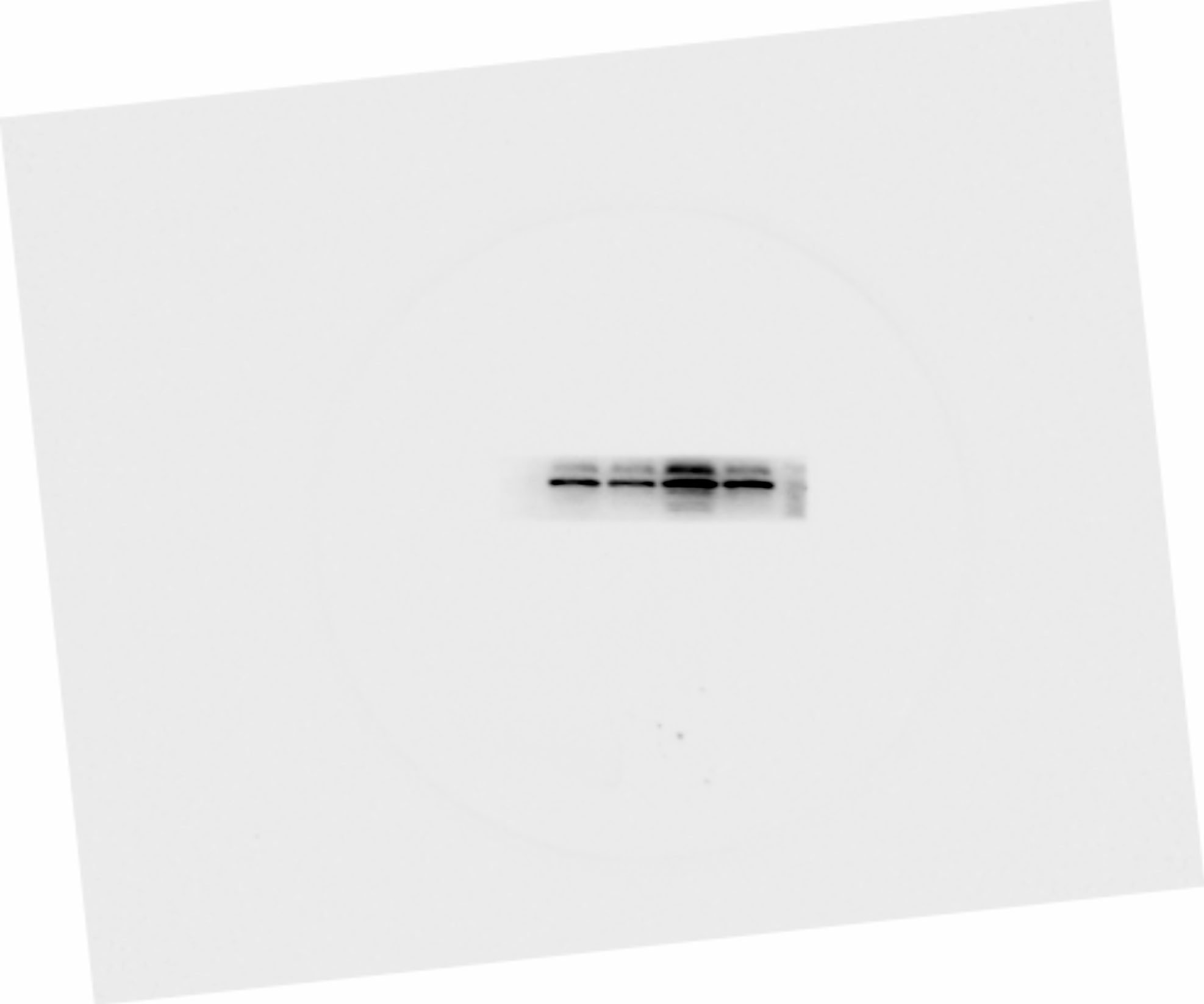

Supplement: S2 File — (ZIP) [file pone.0313803.s002.zip › Uncropped western blots/WB-MDA/SYZX 2024-04-25 12h24m45s.tif]

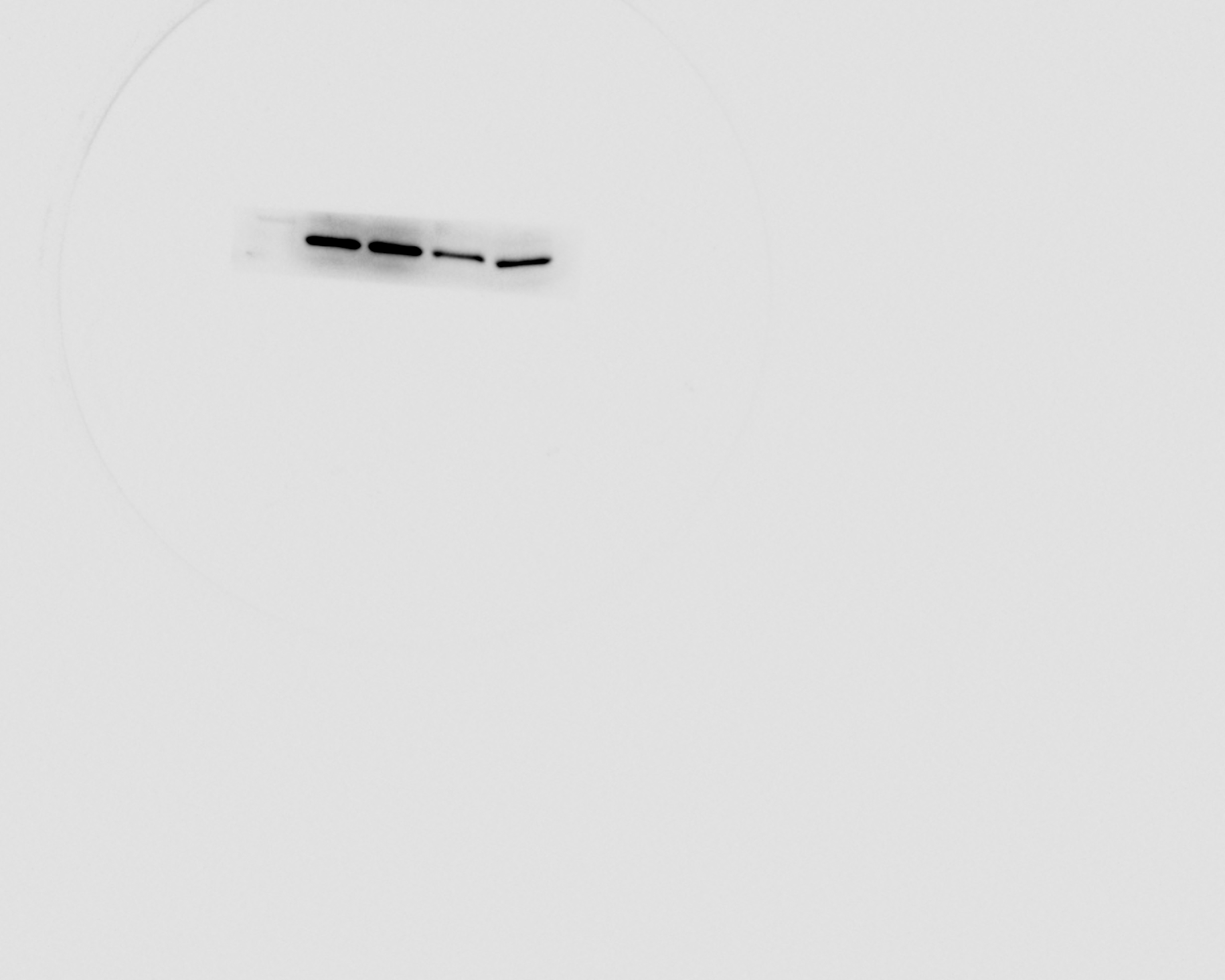

Supplement: S2 File — (ZIP) [file pone.0313803.s002.zip › Uncropped western blots/WB-Nrf2/SYZX 2024-04-17 10h41m10s.jpg]

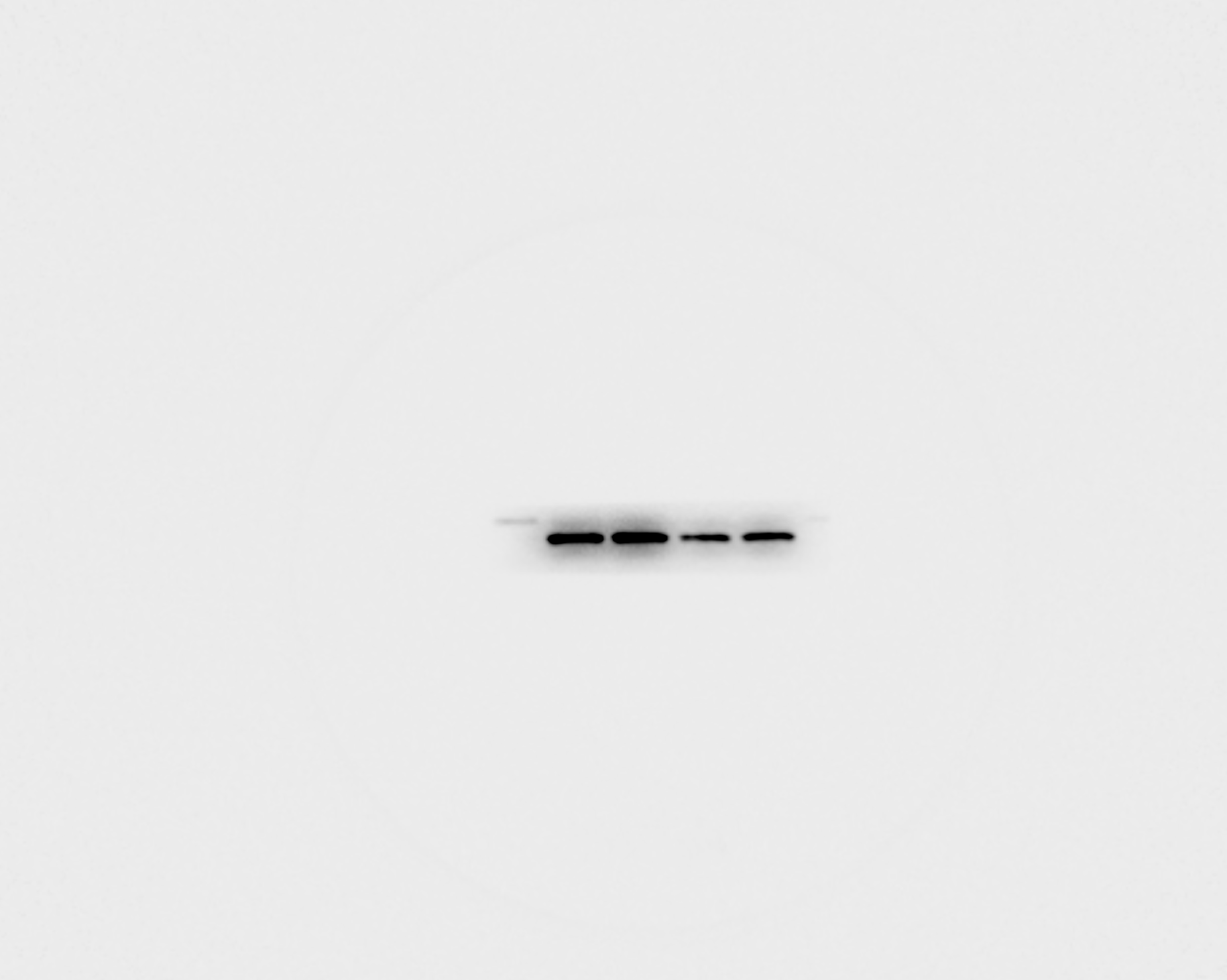

Supplement: S2 File — (ZIP) [file pone.0313803.s002.zip › Uncropped western blots/WB-Nrf2/SYZX 2024-04-18 11h50m38s.tif]

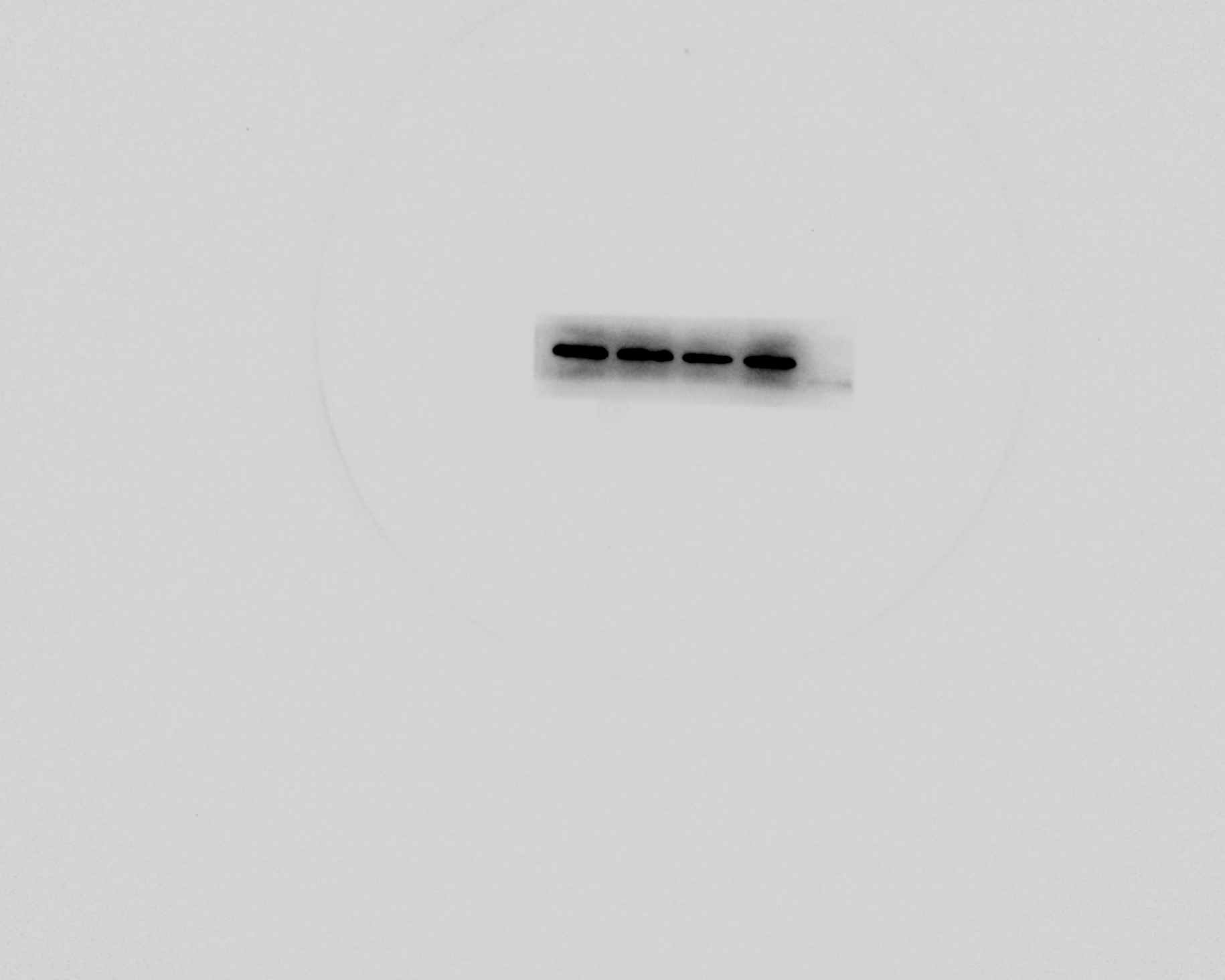

Supplement: S2 File — (ZIP) [file pone.0313803.s002.zip › Uncropped western blots/WB-Nrf2/SYZX 2024-04-25 12h14m01s.jpg]

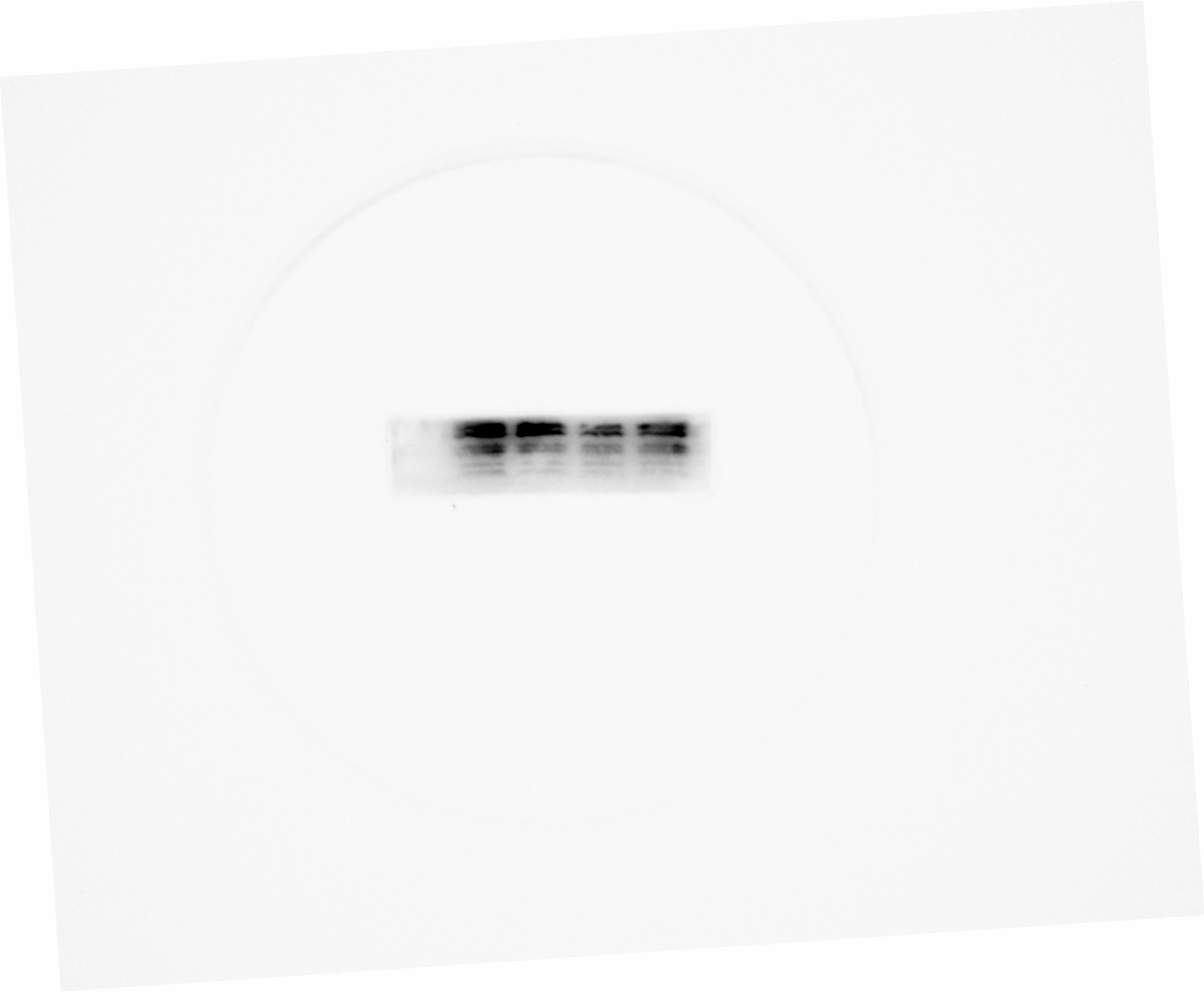

Supplement: S2 File — (ZIP) [file pone.0313803.s002.zip › Uncropped western blots/WB-Sod/SYZX 2024-04-17 10h20m30s.tif]

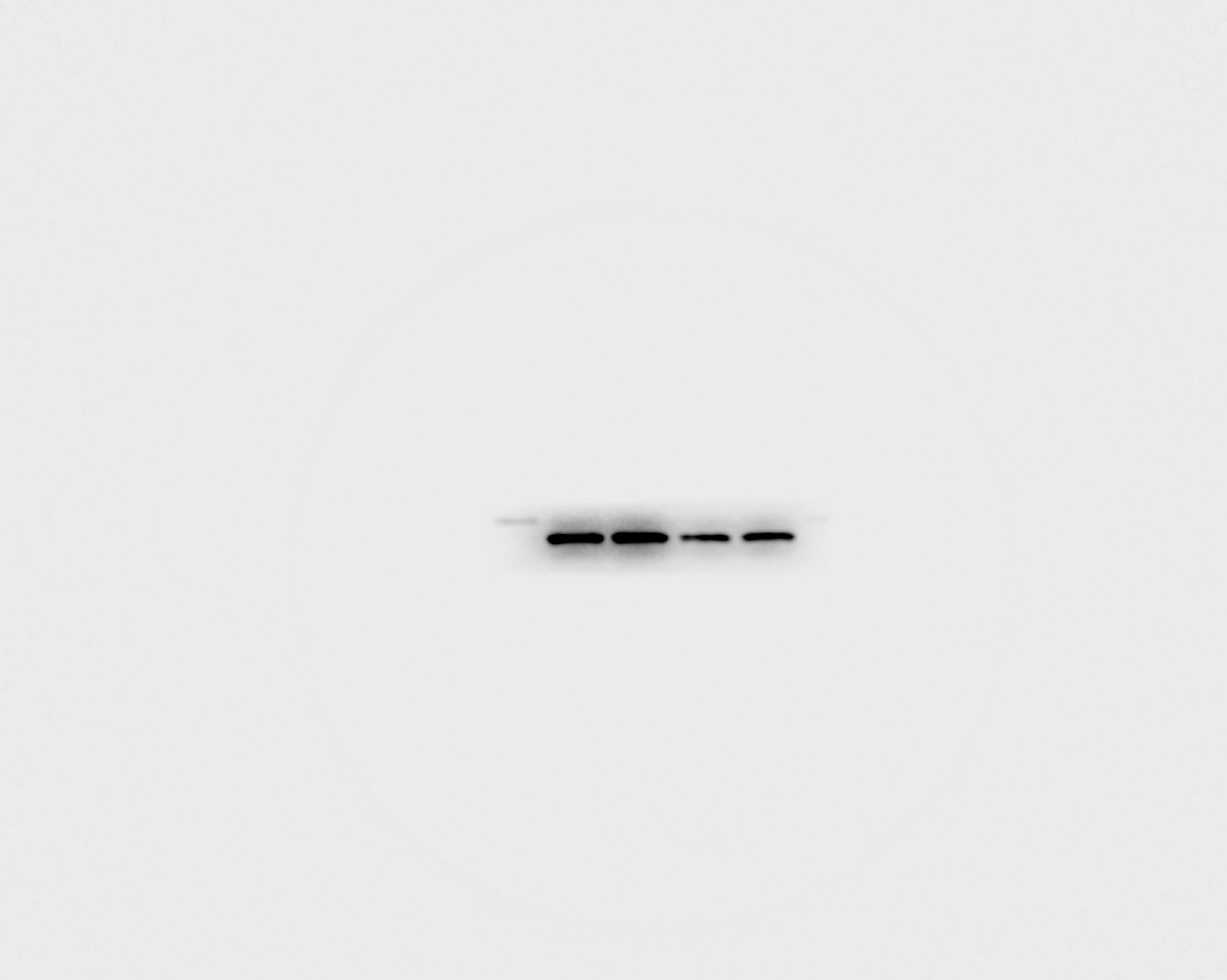

Supplement: S2 File — (ZIP) [file pone.0313803.s002.zip › Uncropped western blots/WB-Sod/SYZX 2024-04-18 12h05m06s.jpg]

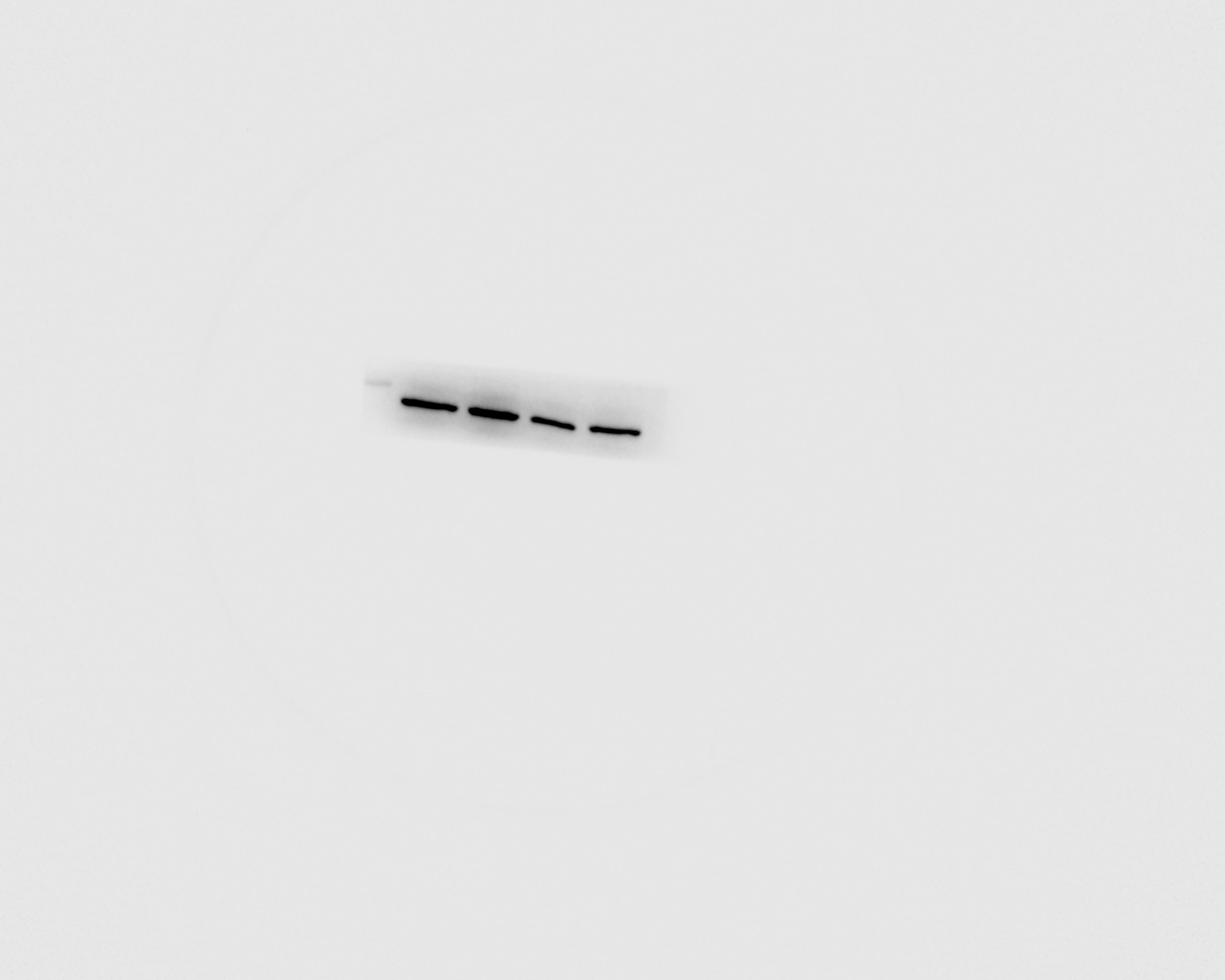

Supplement: S2 File — (ZIP) [file pone.0313803.s002.zip › Uncropped western blots/WB-Sod/SYZX 2024-04-25 12h28m25s.jpg]

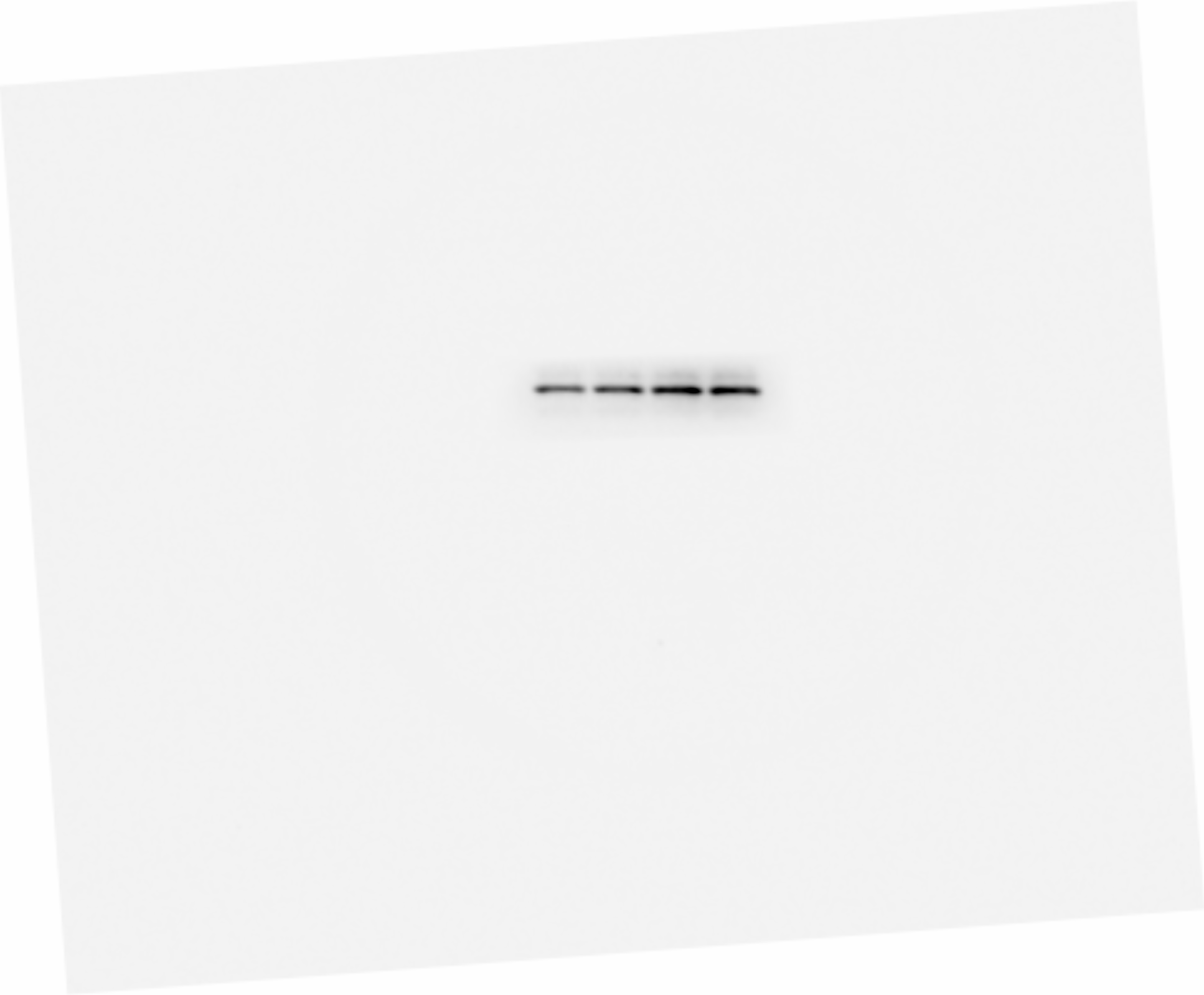

Supplement: S2 File — (ZIP) [file pone.0313803.s002.zip › Uncropped western blots/WB-caspase-3/SYZX 2024-04-17 10h12m54s.tif]

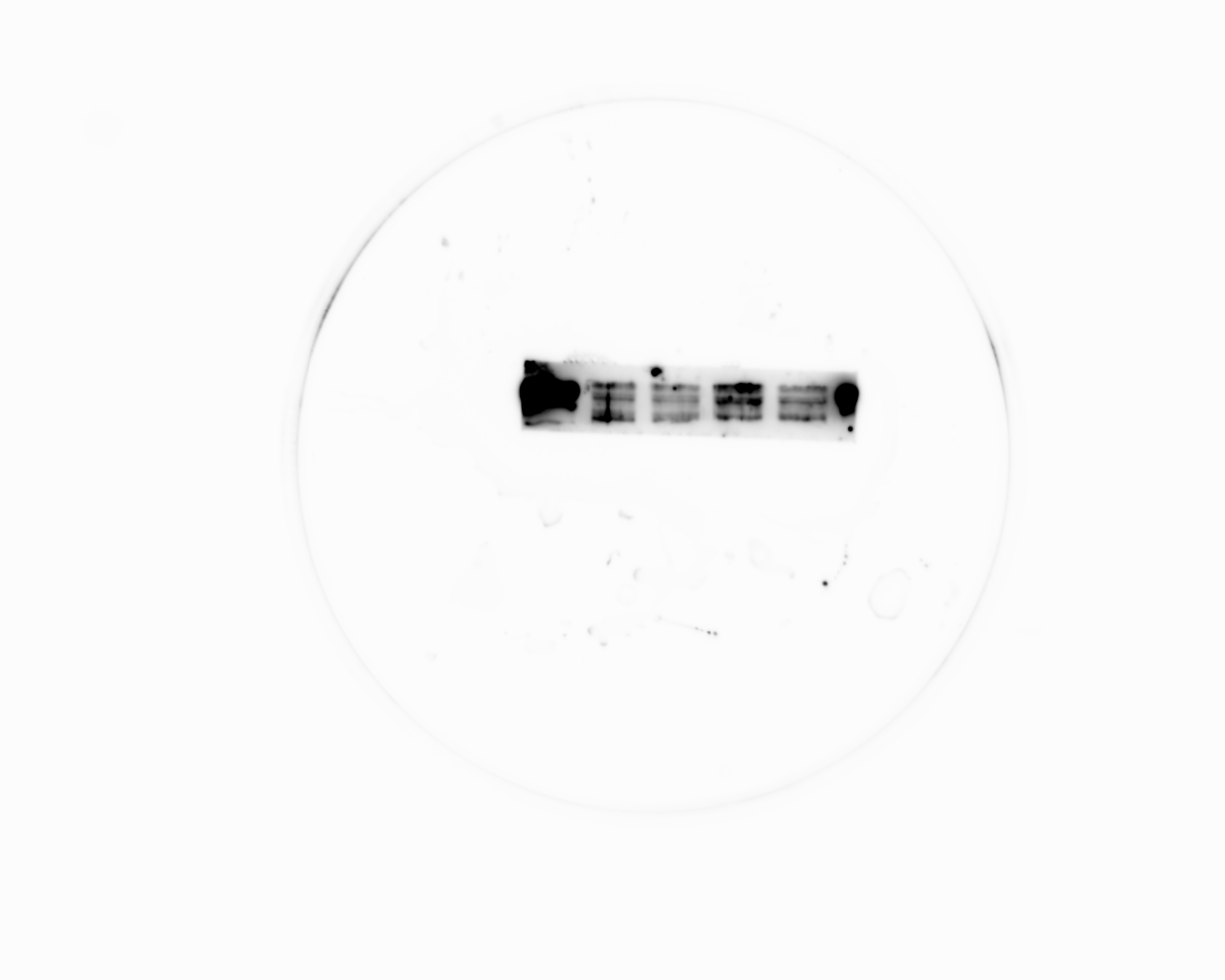

Supplement: S2 File — (ZIP) [file pone.0313803.s002.zip › Uncropped western blots/WB-caspase-3/SYZX 2024-04-17 10h15m22s.tif]

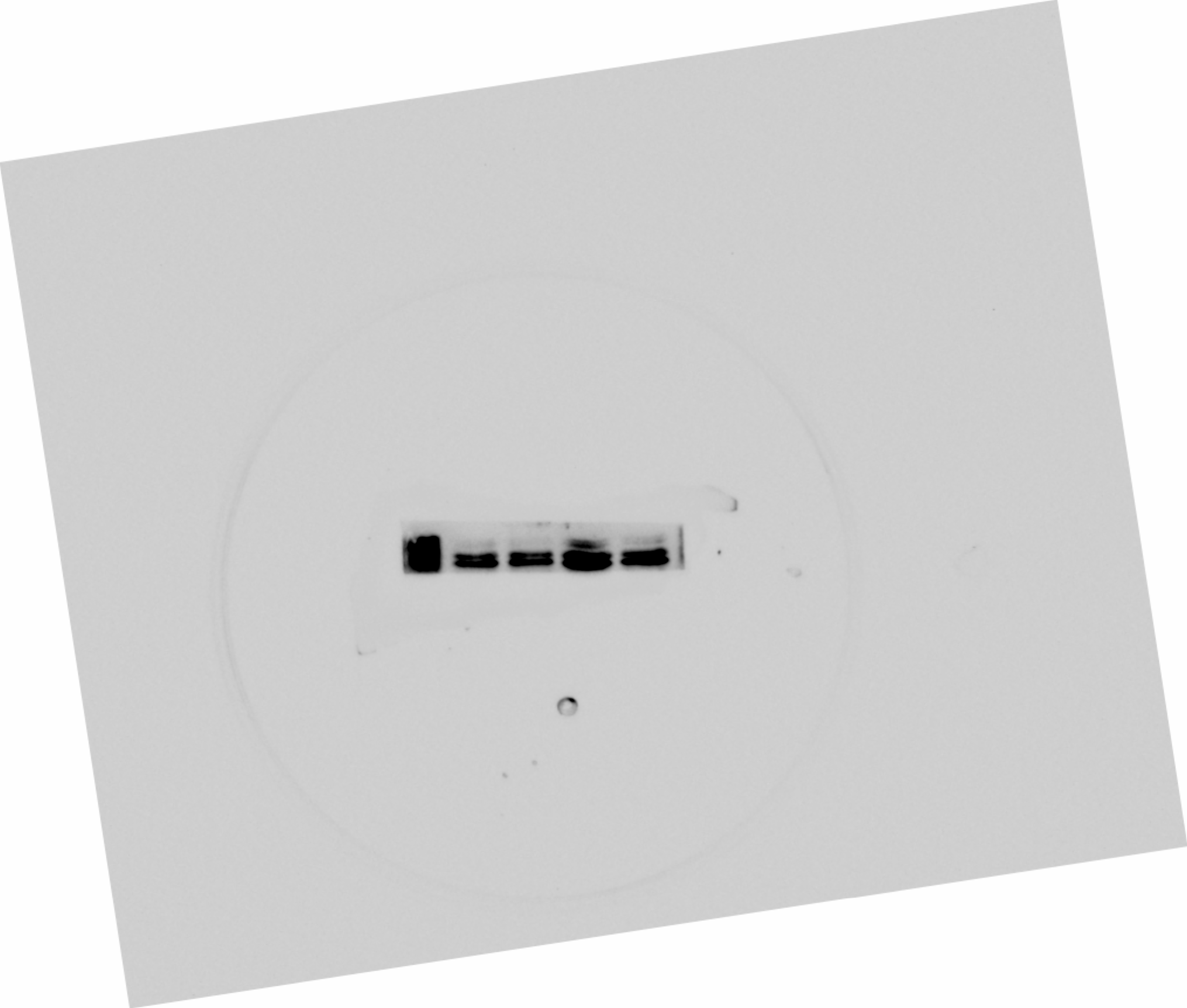

Supplement: S2 File — (ZIP) [file pone.0313803.s002.zip › Uncropped western blots/WB-caspase-3/SYZX 2024-04-18 12h05m11s.tif]

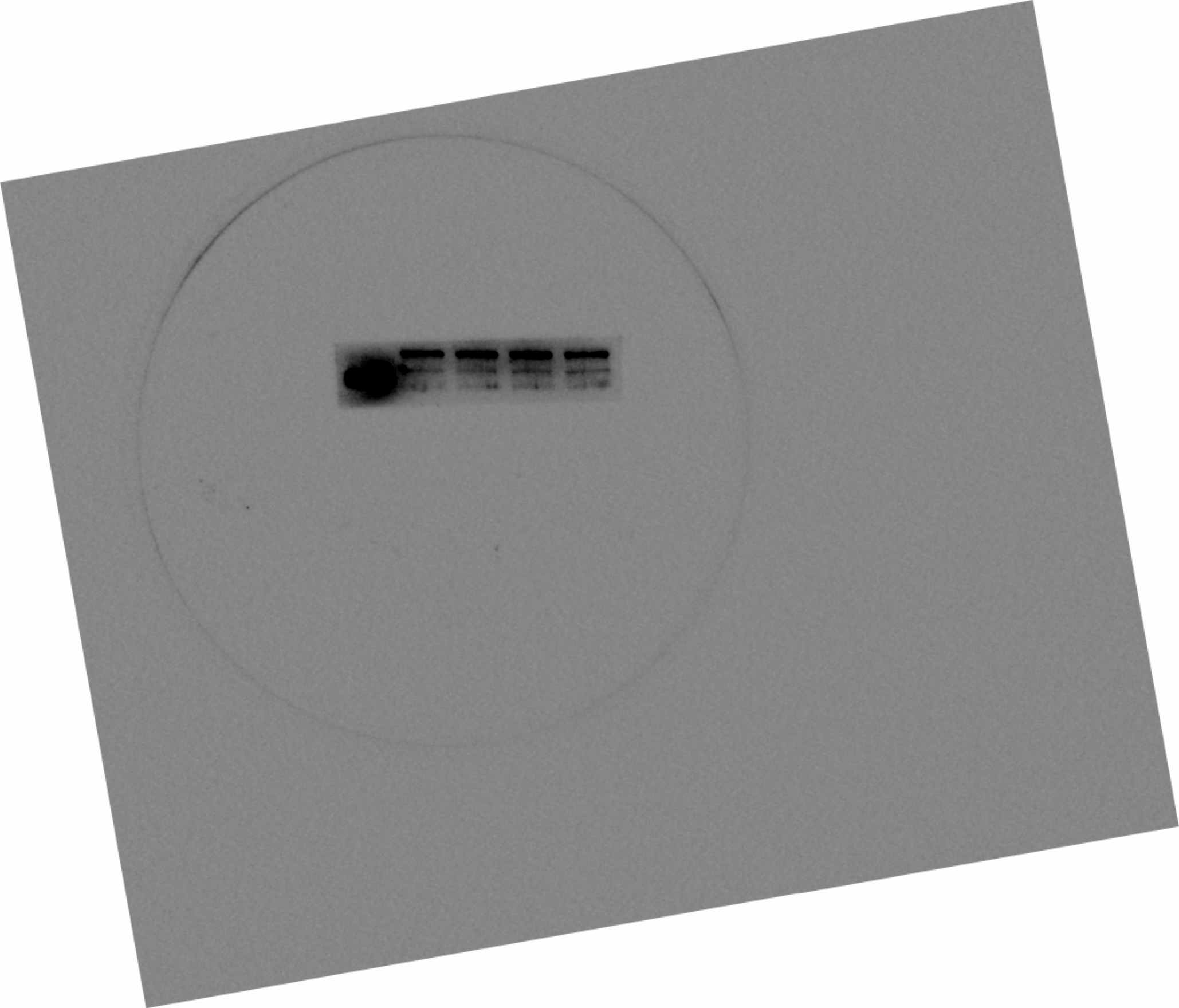

Supplement: S2 File — (ZIP) [file pone.0313803.s002.zip › Uncropped western blots/WB-caspase-3/SYZX 2024-04-25 12h20m38s.tif]

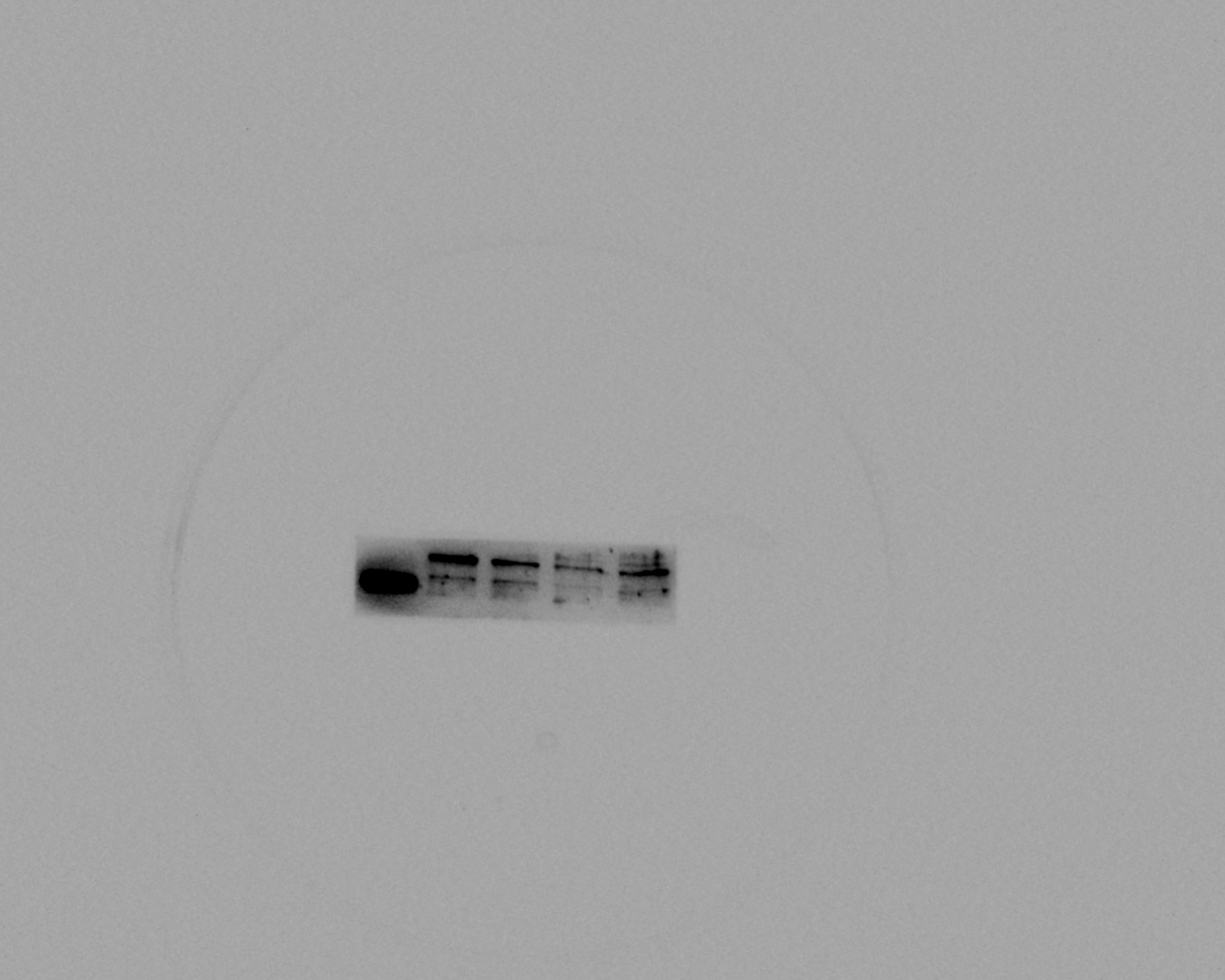

Supplement: S2 File — (ZIP) [file pone.0313803.s002.zip › Uncropped western blots/WB-chop/SYZX 2024-04-17 10h40m13s.jpg]

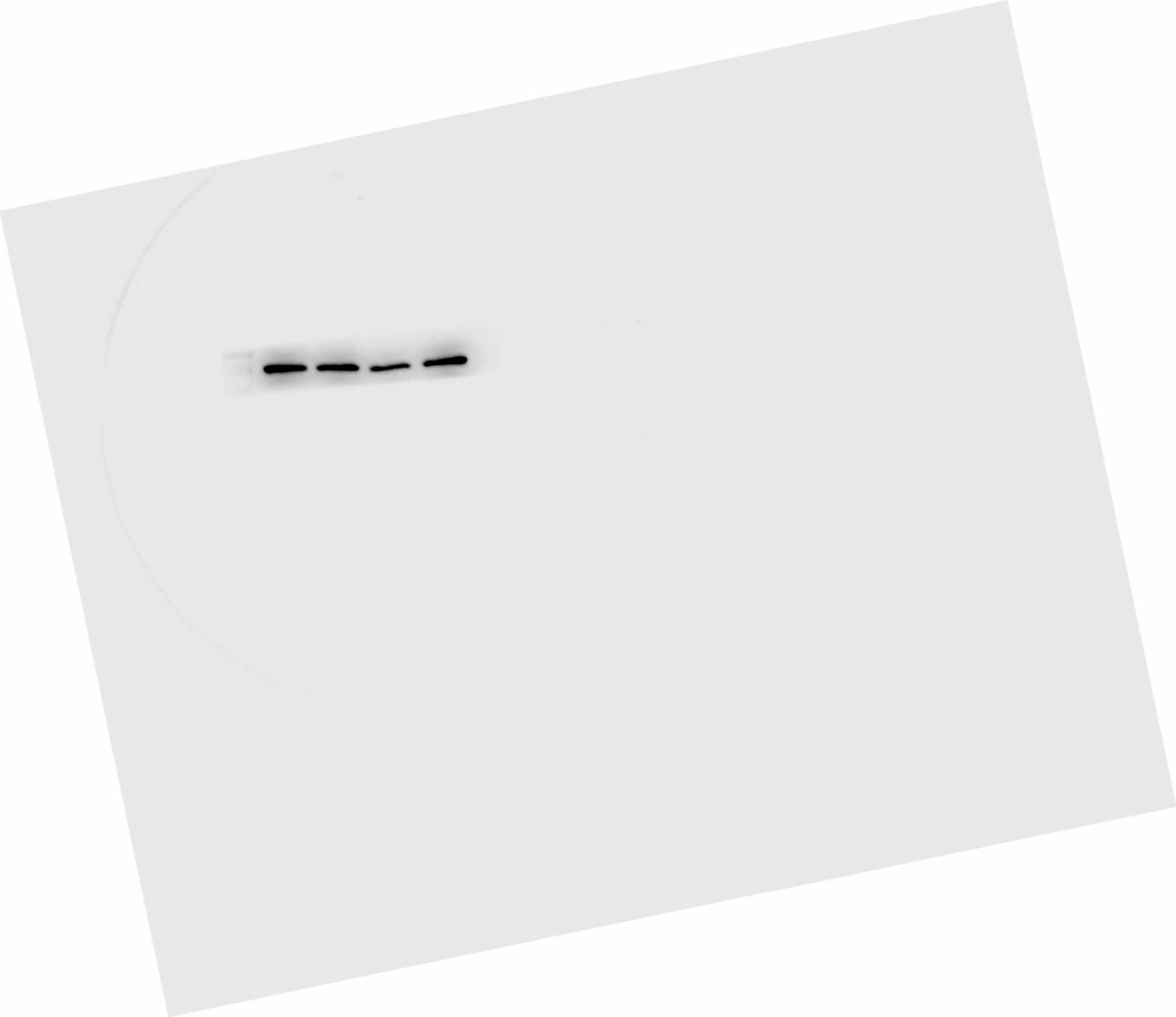

Supplement: S2 File — (ZIP) [file pone.0313803.s002.zip › Uncropped western blots/WB-chop/SYZX 2024-04-18 11h57m45s.tif]

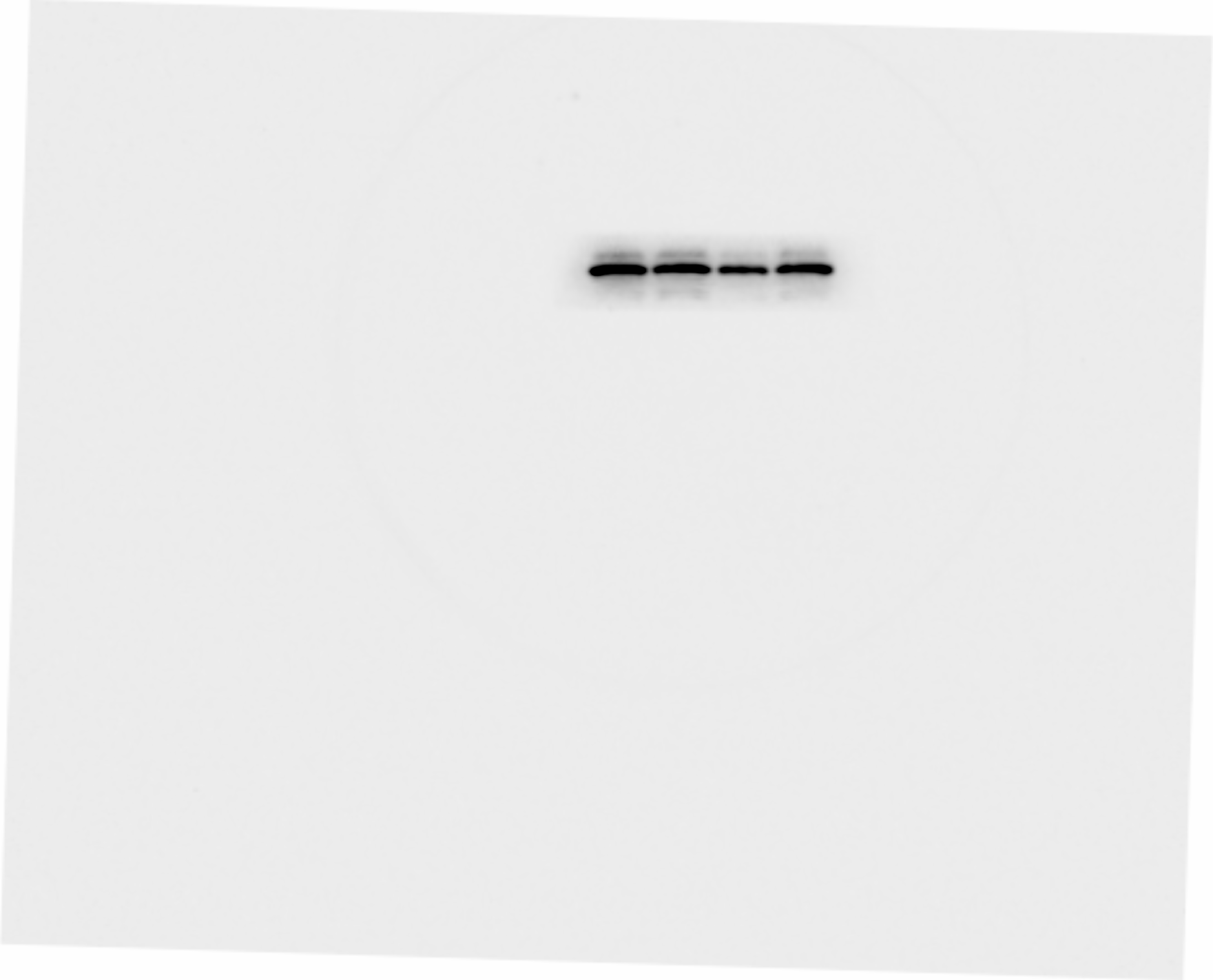

Supplement: S2 File — (ZIP) [file pone.0313803.s002.zip › Uncropped western blots/WB-chop/SYZX 2024-04-25 12h15m59s.tif]

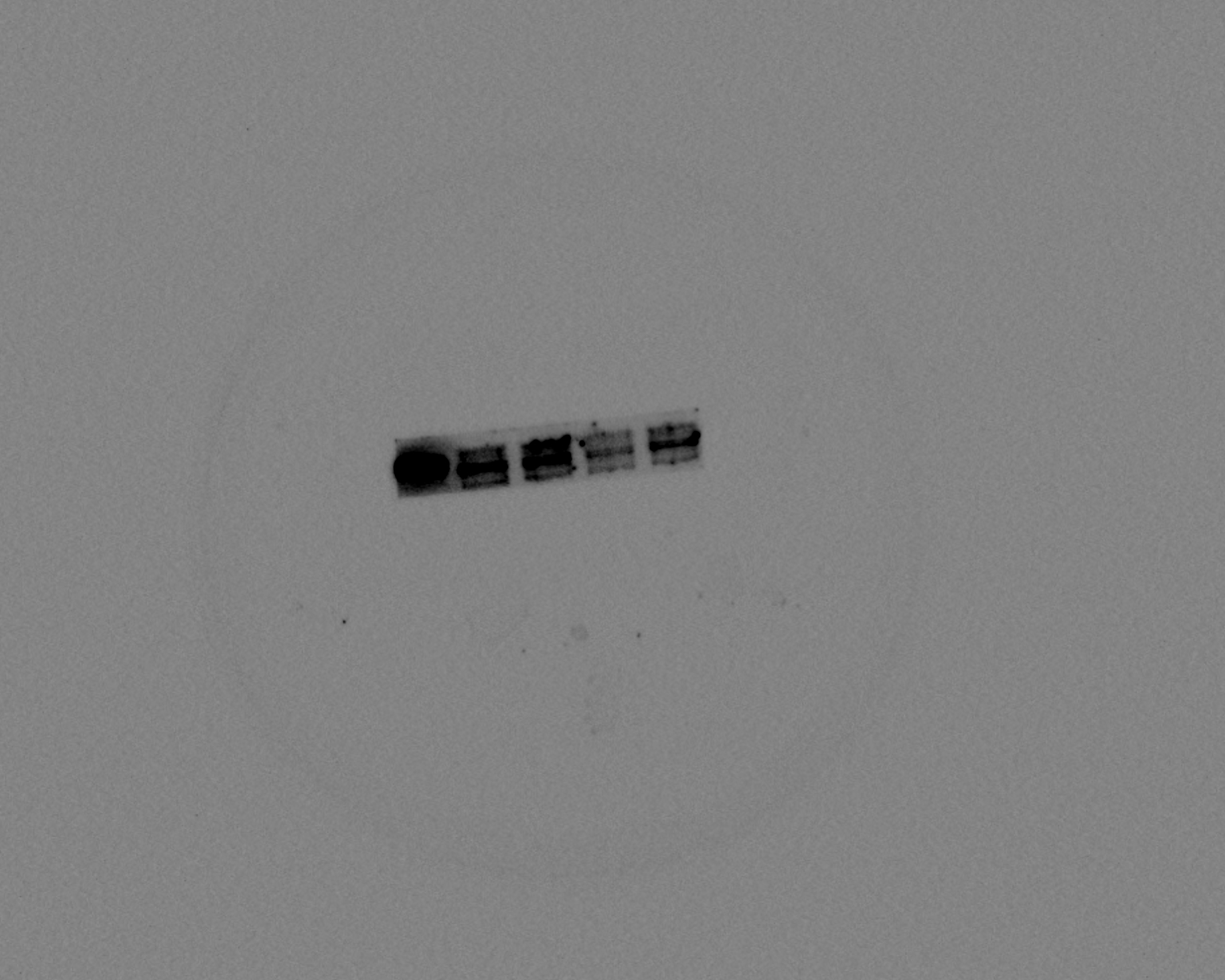

Supplement: S2 File — (ZIP) [file pone.0313803.s002.zip › Uncropped western blots/WB-hsp60/SYZX 2024-04-17 10h38m27s.jpg]

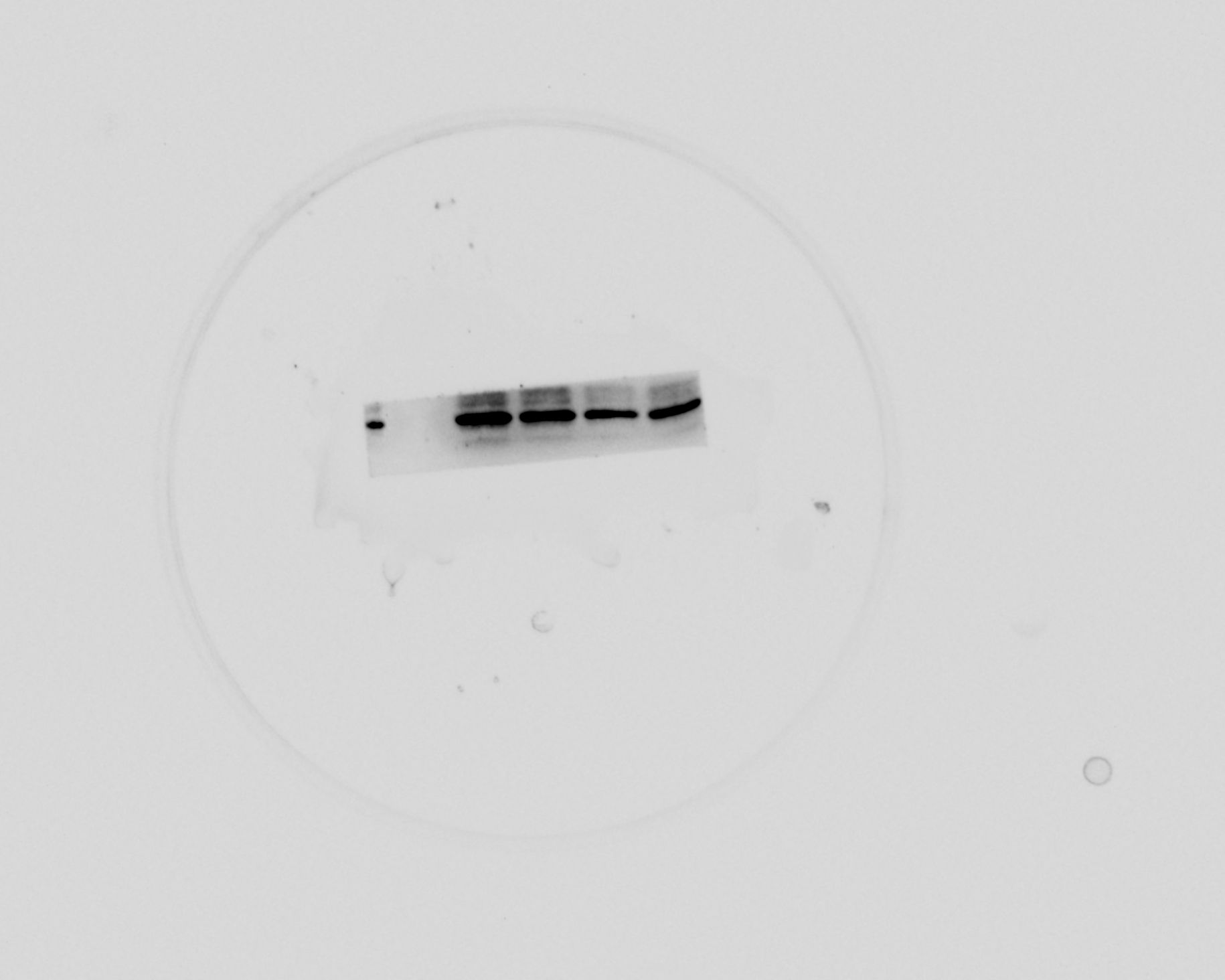

Supplement: S2 File — (ZIP) [file pone.0313803.s002.zip › Uncropped western blots/WB-hsp60/SYZX 2024-04-18 11h43m23s.jpg]

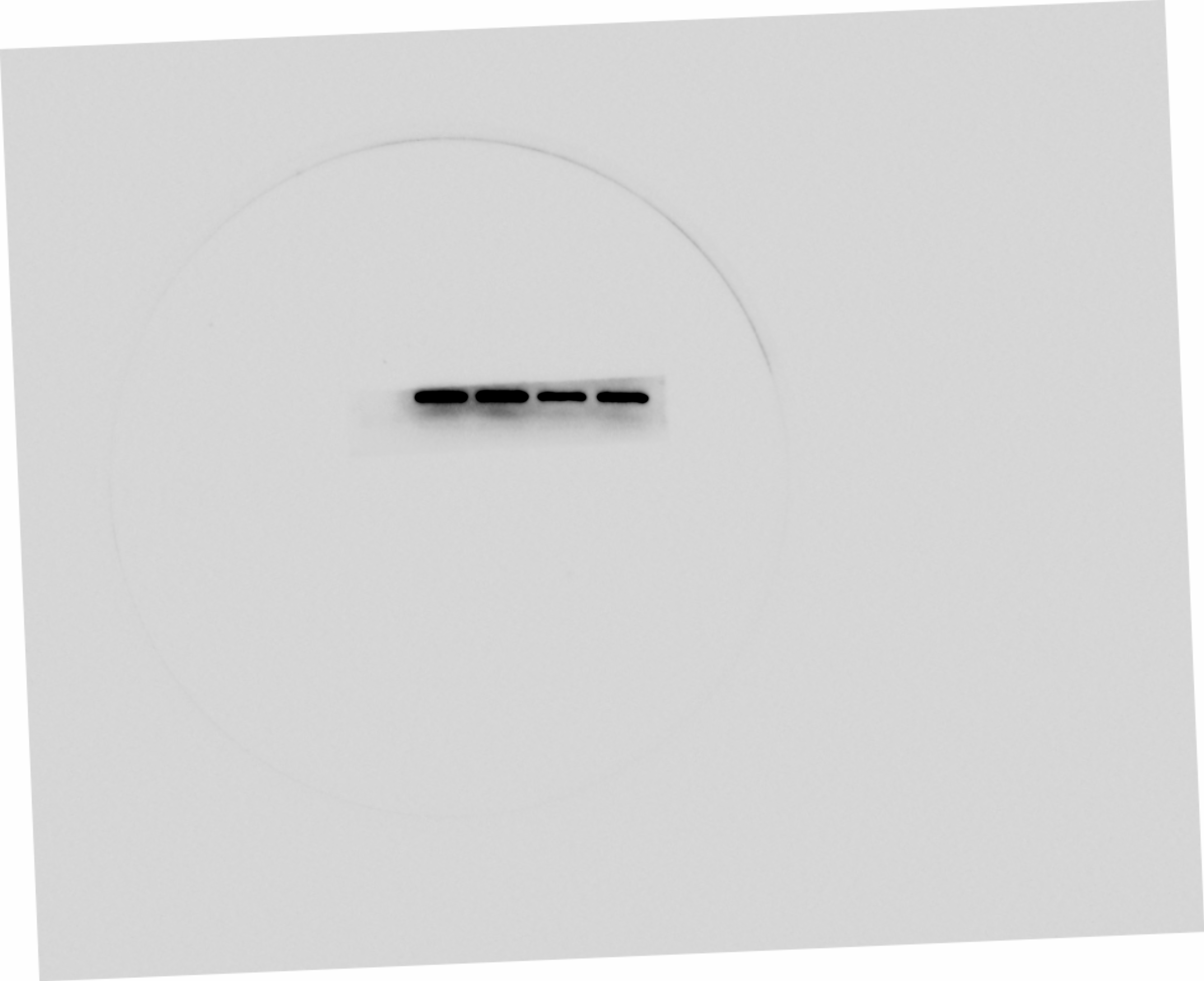

Supplement: S2 File — (ZIP) [file pone.0313803.s002.zip › Uncropped western blots/WB-hsp60/SYZX 2024-04-25 12h20m33s.tif]

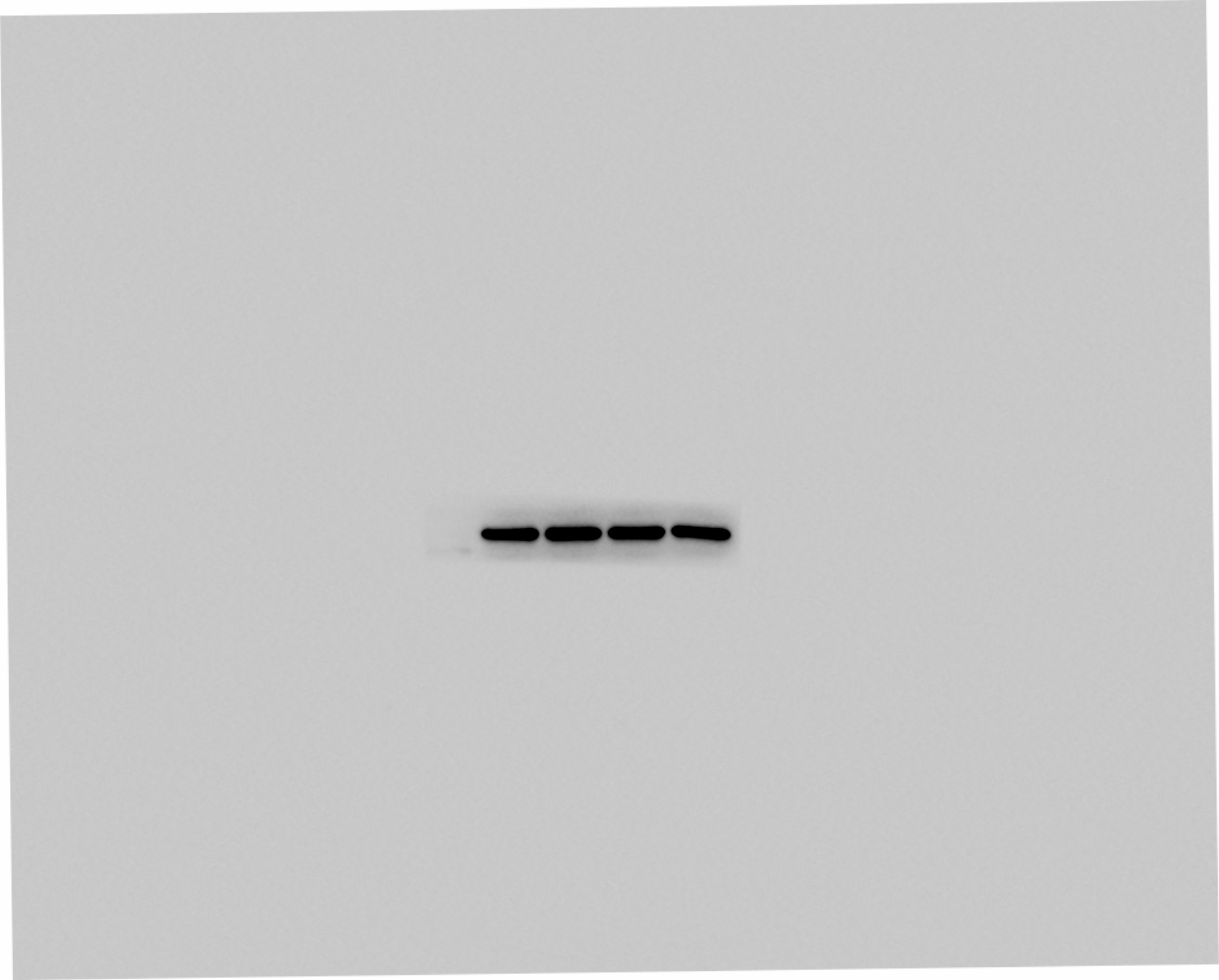

Supplement: S2 File — (ZIP) [file pone.0313803.s002.zip › Uncropped western blots/WB-βactin/SYZX 2024-04-17 10h10m58s.tif]

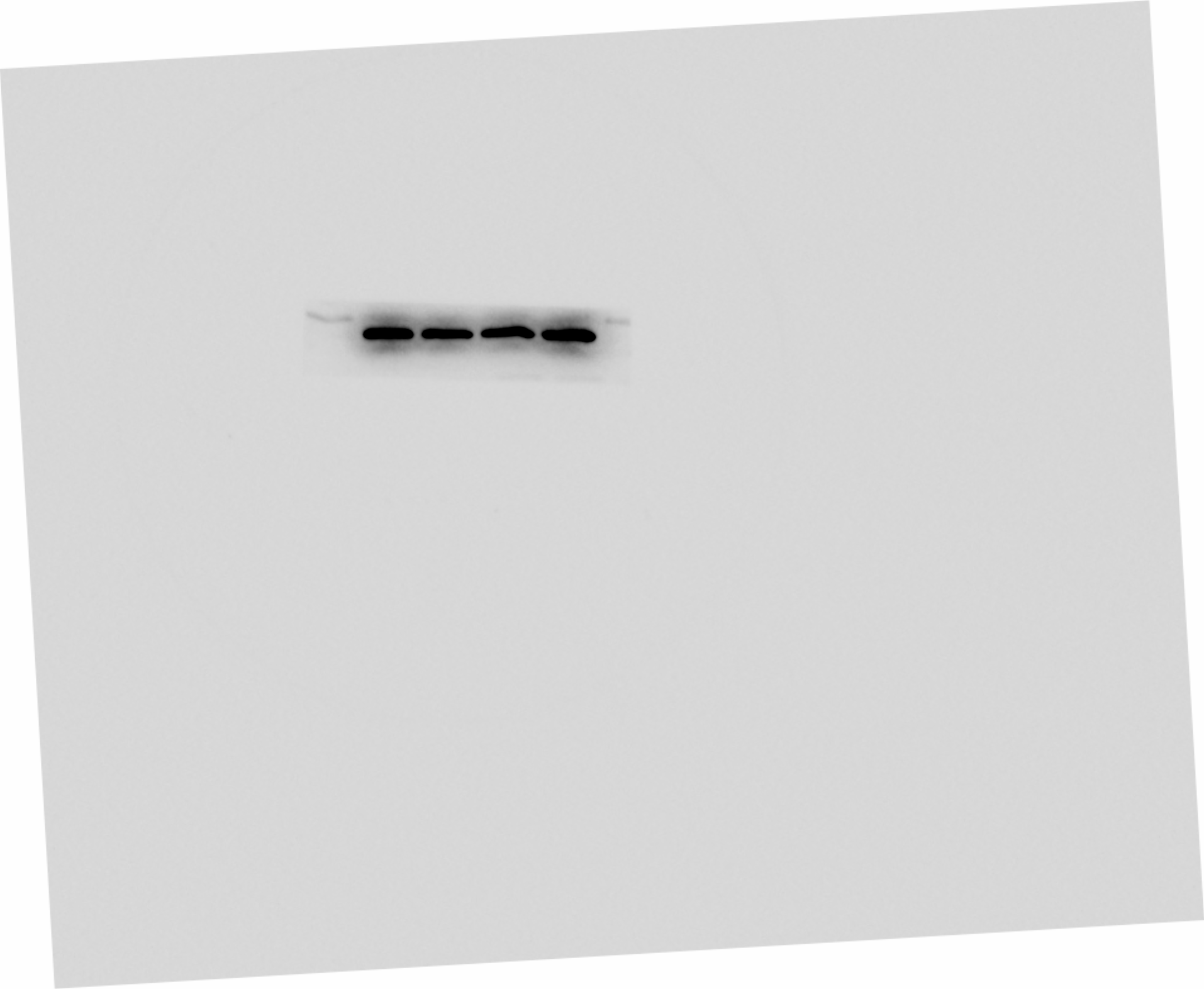

Supplement: S2 File — (ZIP) [file pone.0313803.s002.zip › Uncropped western blots/WB-βactin/SYZX 2024-04-17 10h40m02s.tif]

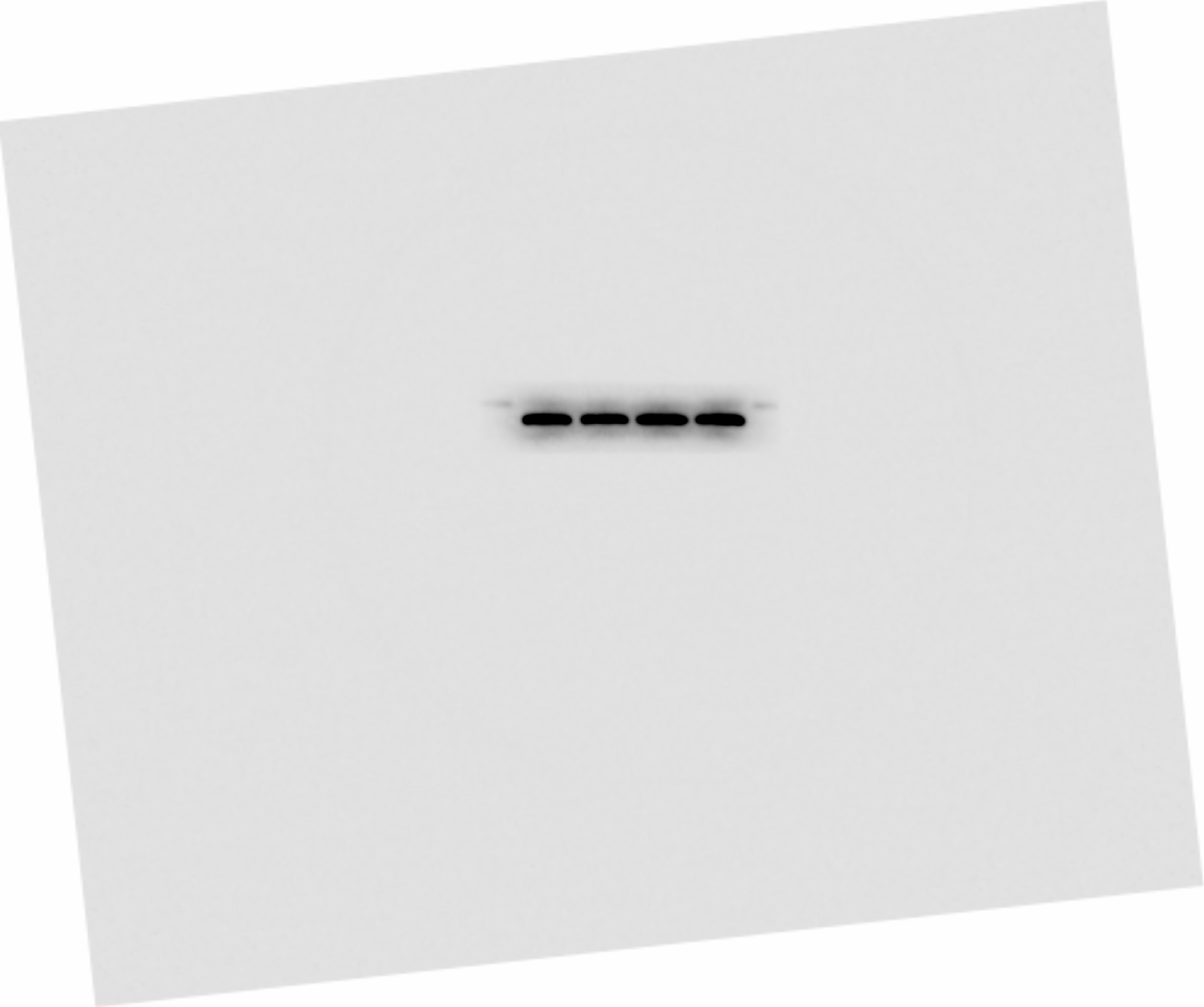

Supplement: S2 File — (ZIP) [file pone.0313803.s002.zip › Uncropped western blots/WB-βactin/SYZX 2024-04-18 11h35m19s.tif]

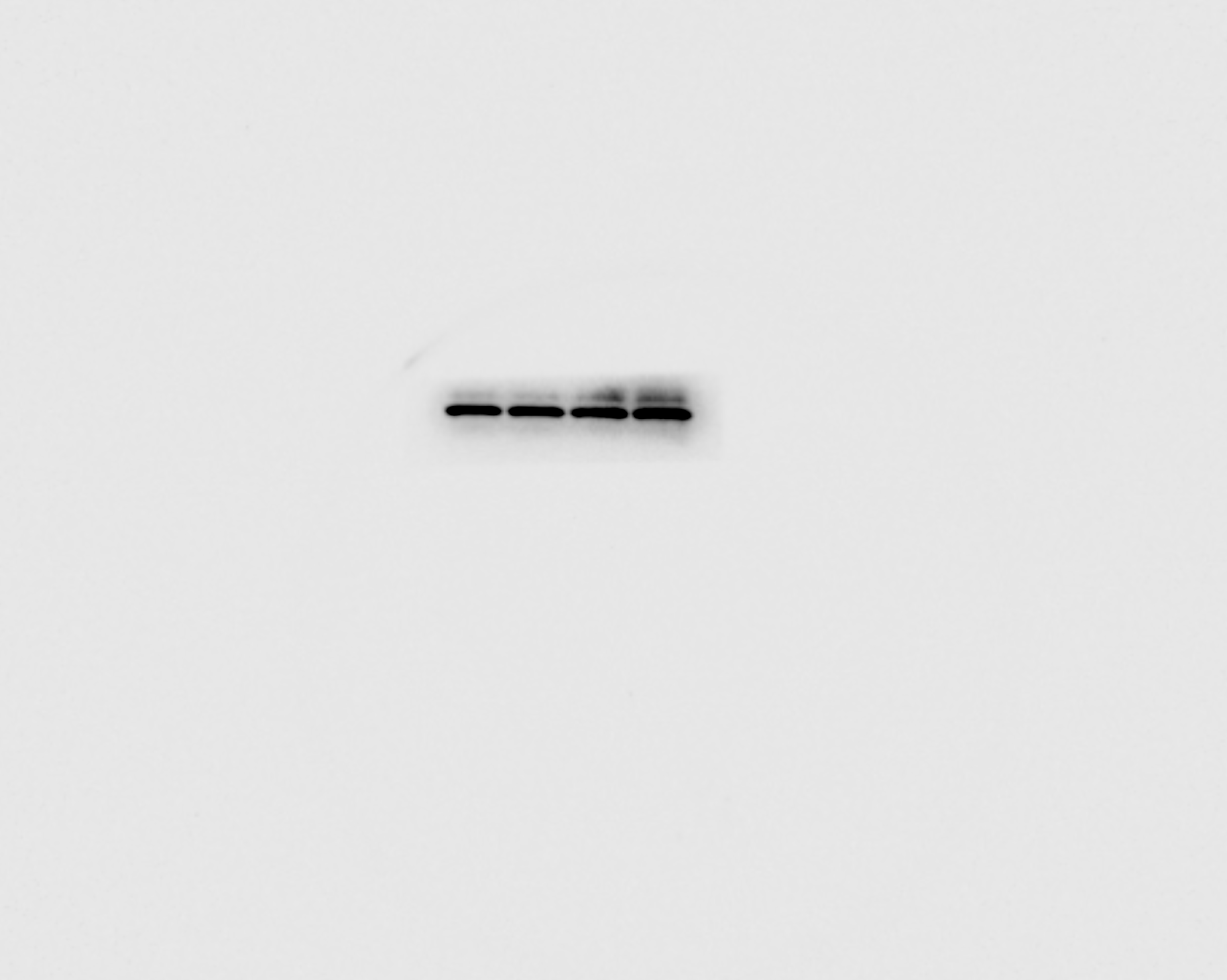

Supplement: S2 File — (ZIP) [file pone.0313803.s002.zip › Uncropped western blots/WB-βactin/SYZX 2024-04-18 12h01m03s.tif]

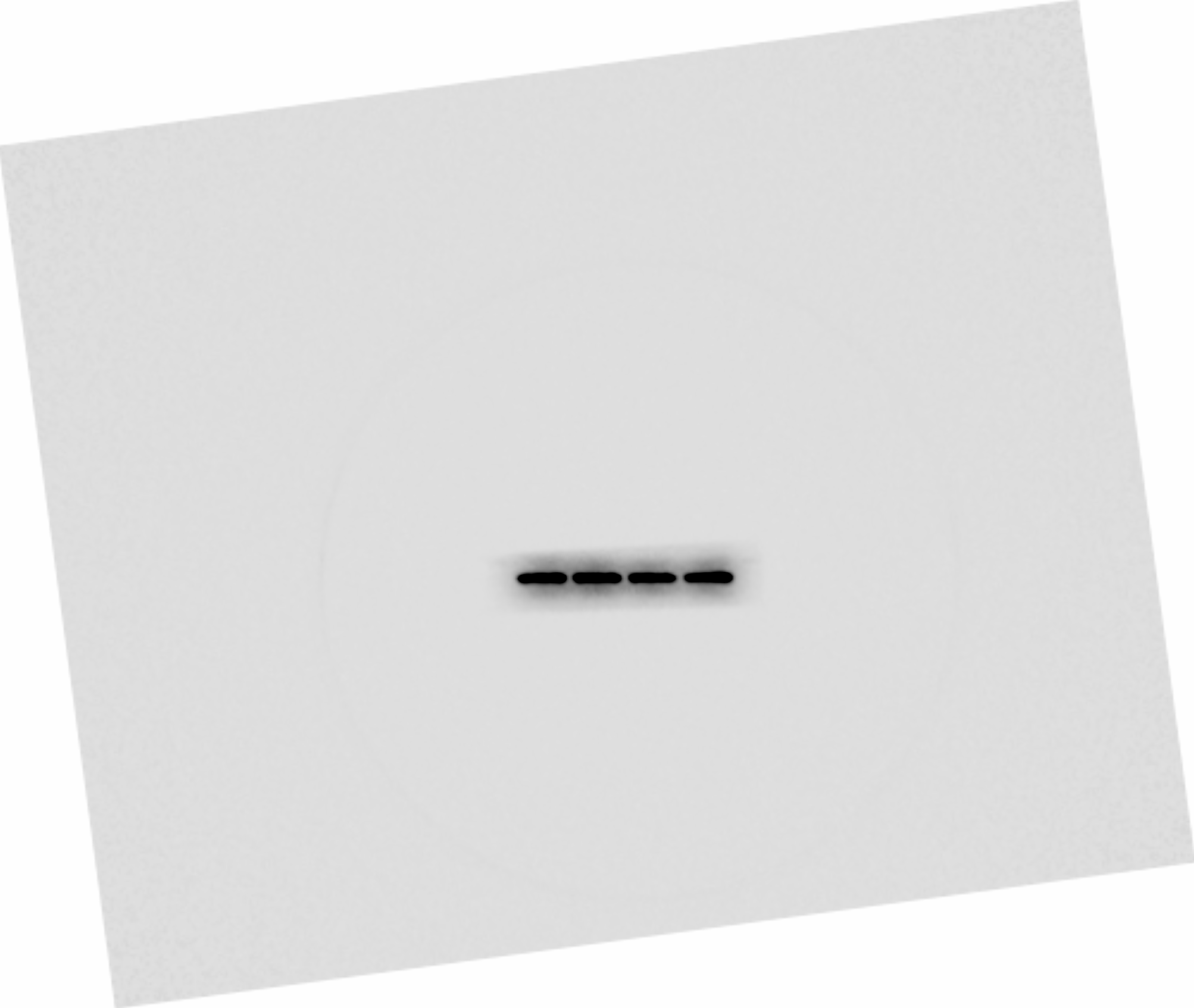

Supplement: S2 File — (ZIP) [file pone.0313803.s002.zip › Uncropped western blots/WB-βactin/SYZX 2024-04-25 12h08m48s.tif]

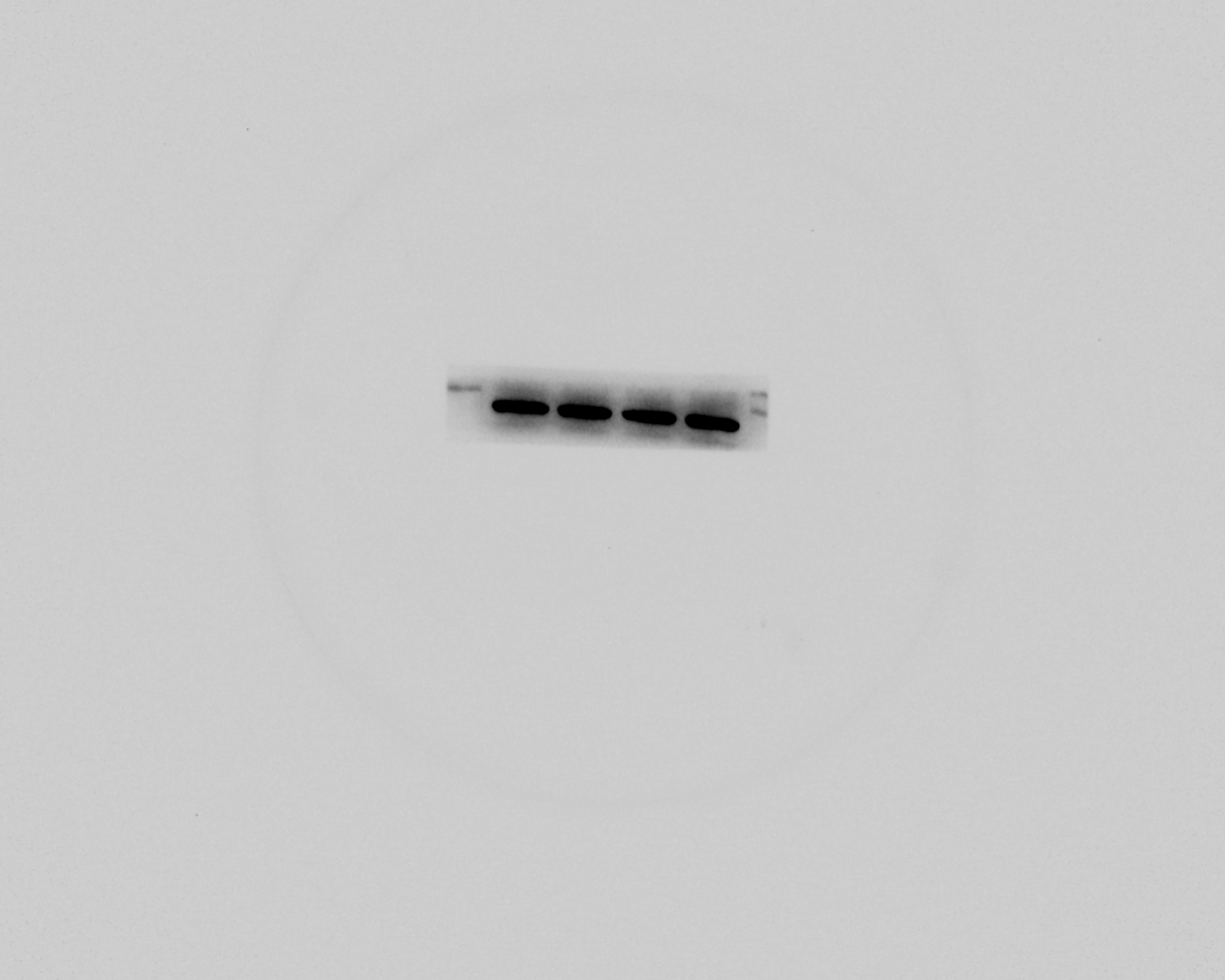

Supplement: S2 File — (ZIP) [file pone.0313803.s002.zip › Uncropped western blots/WB-βactin/SYZX 2024-04-25 12h26m06s.jpg]

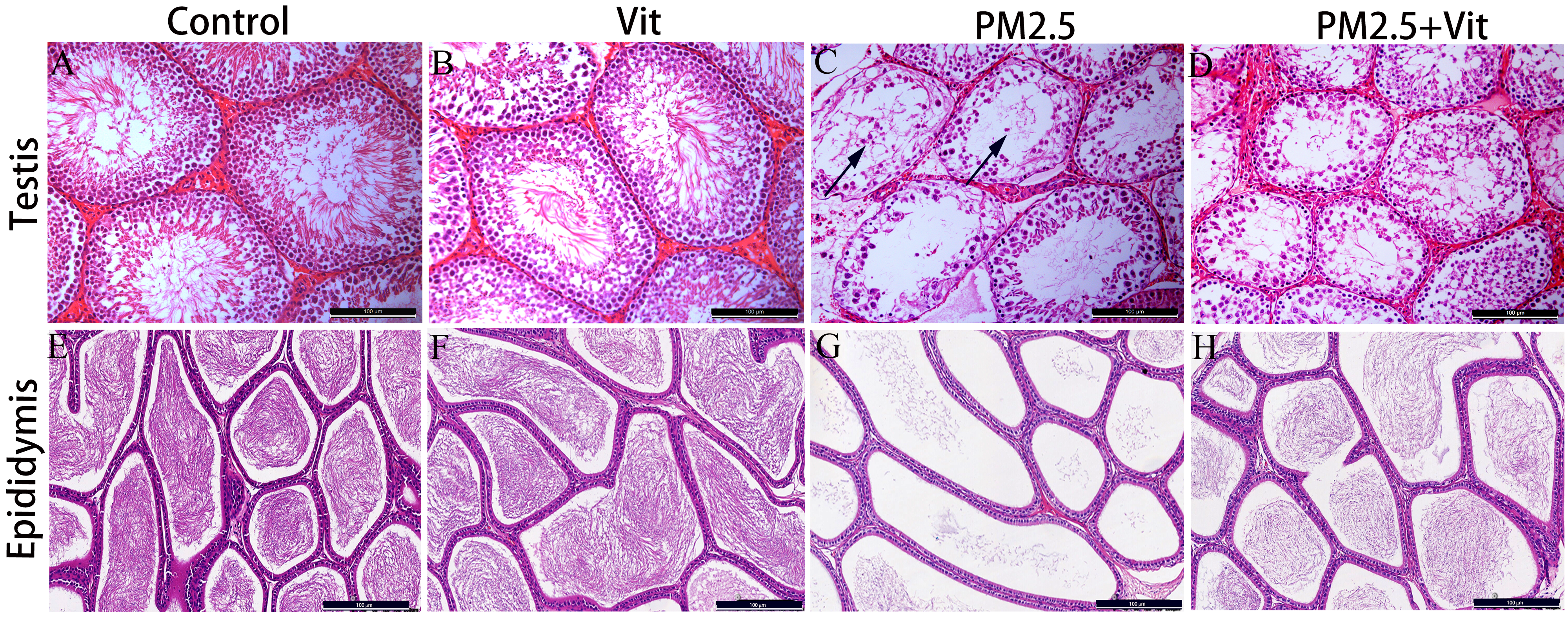

Supplement: S3 File — (ZIP) [file pone.0313803.s003.zip › S3/Morphological changes in the testis and epididymis/Morphological changes in the testis and epididymis.tif]

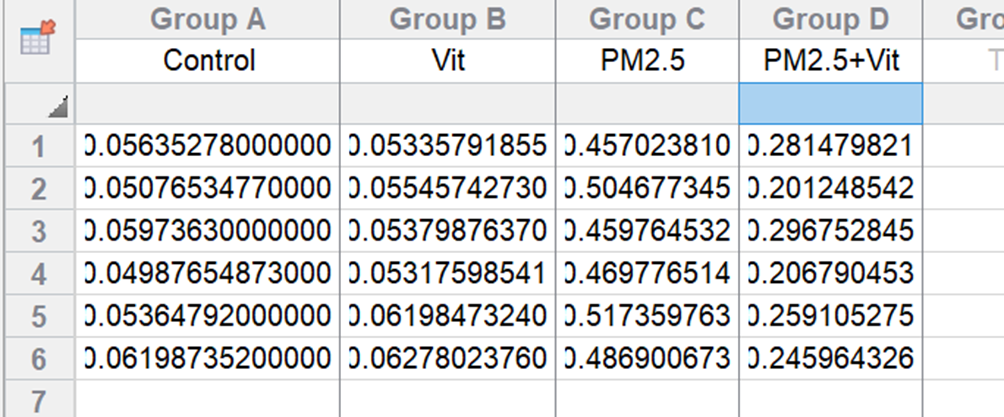


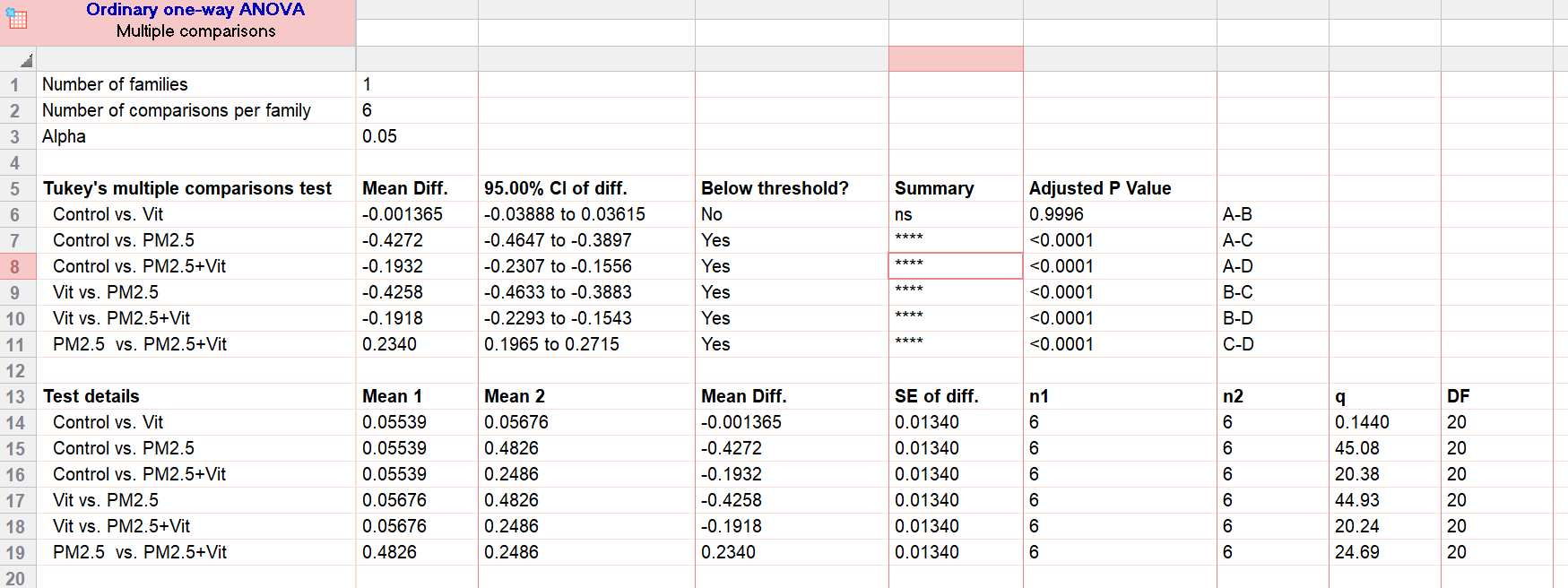

Supplement: S3 File — (ZIP) [file pone.0313803.s003.zip › S3/TUNEL staining of testis tissues/data.docx]

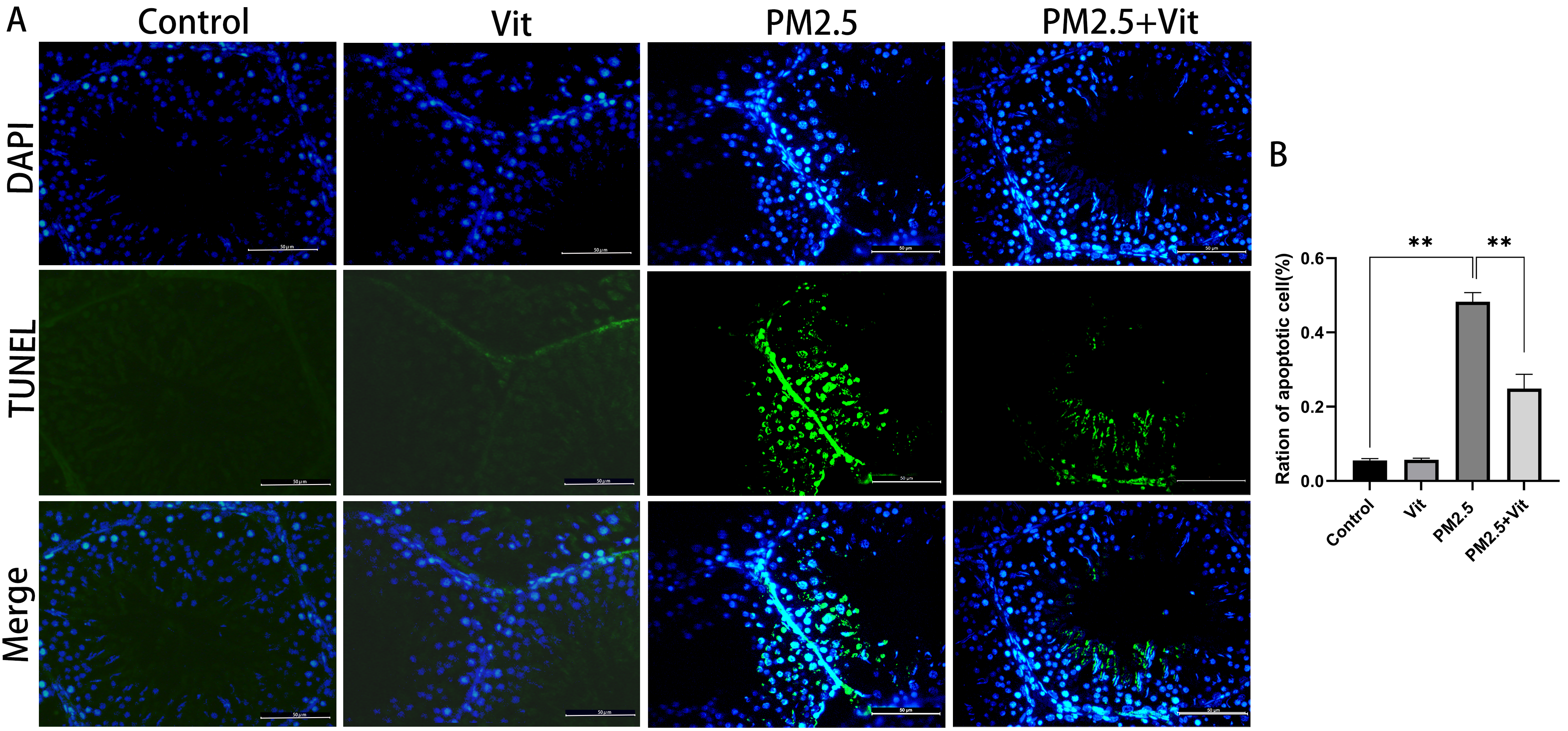

Supplement: S3 File — (ZIP) [file pone.0313803.s003.zip › S3/TUNEL staining of testis tissues/TUNEL staining of testis tissues.tif]

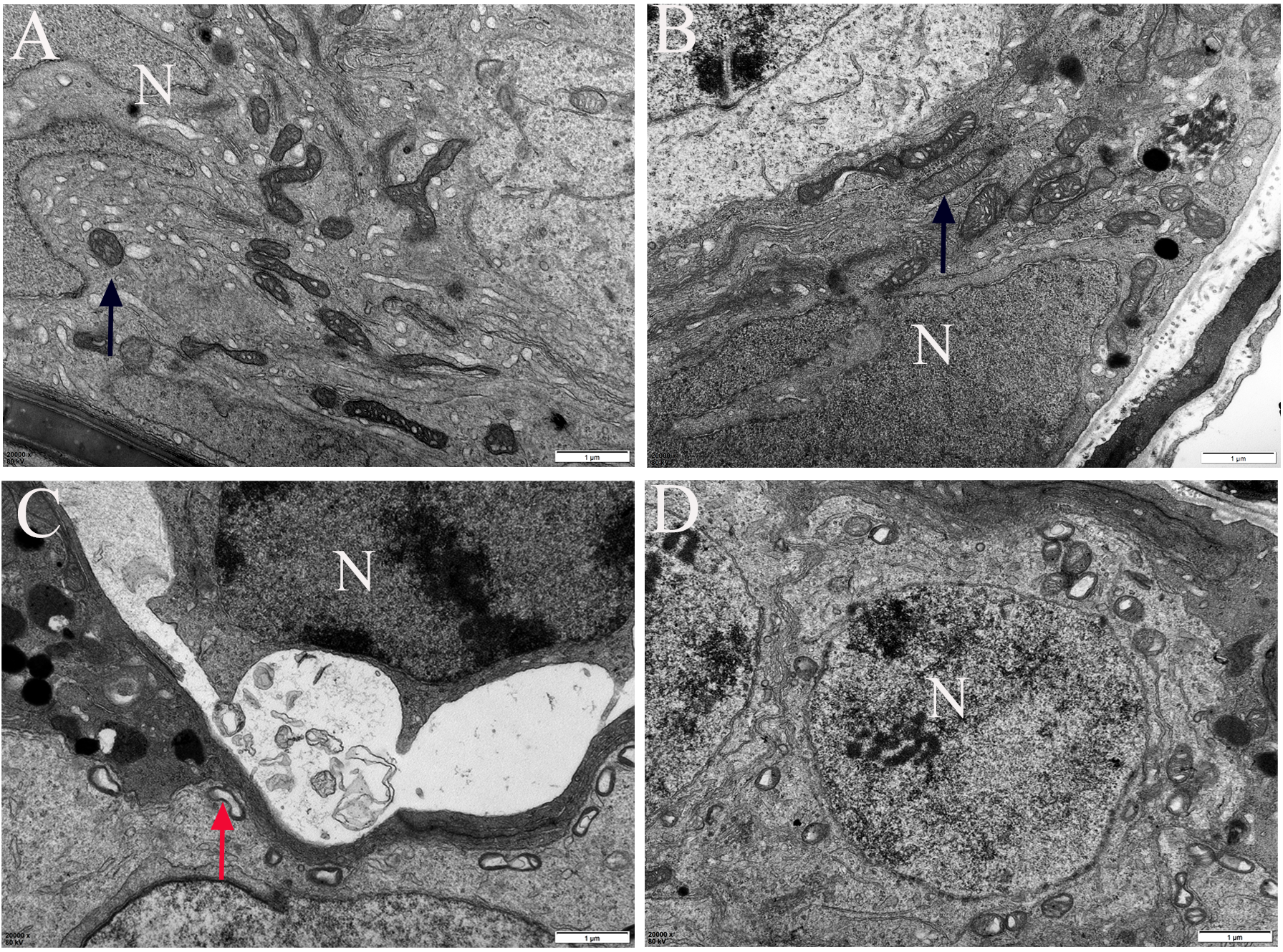

Supplement: S3 File — (ZIP) [file pone.0313803.s003.zip › S3/Ultrastructure of mitochondria in germ cells/Ultrastructure of mitochondria in germ cells.tif]
